# 4‐Sulfamoylphenylalkylamides as Inhibitors of Carbonic Anhydrases Expressed in *Vibrio cholerae*

**DOI:** 10.1002/cmdc.202100510

**Published:** 2021-10-18

**Authors:** Francesca Mancuso, Laura De Luca, Federica Bucolo, Milan Vrabel, Andrea Angeli, Clemente Capasso, Claudiu T. Supuran, Rosaria Gitto

**Affiliations:** ^1^ CHIBIOFARAM Department University of Messina Viale Stagno D'Alcontres 98166 Messina Italy; ^2^ Institute of Organic Chemistry and Biochemistry (IOCB) Czech Academy of Sciences Flemingovo nám. 2 16000 Prague Czech Republic; ^3^ NEUROFARBA Department University of Florence Via U. Schiff 6 50019 Florence Italy; ^4^ Institute of Biosciences and Bioresources CNR Via Castellino 111 80131 Napoli Italy

**Keywords:** Drug Discovery, Sulfonamides, Enzymes Inhibitors, Vibrio cholerae, Bacterial carbonic anhydrases

## Abstract

A current issue of antimicrobial therapy is the resistance to treatment with worldwide consequences. Thus, the identification of innovative targets is an intriguing challenge in the drug and development process aimed at newer antimicrobial agents. The state‐of‐art of anticholera therapy might comprise the reduction of the expression of cholera toxin, which could be reached through the inhibition of carbonic anhydrases expressed in *Vibrio cholerae* (VchCAα, VchCAβ, and VchCAγ). Therefore, we focused our interest on the exploitation of sulfonamides as VchCA inhibitors. We planned to design and synthesize new benzenesulfonamides based on our knowledge of the VchCA catalytic site. The synthesized compounds were tested thus collecting useful SAR information. From our investigation, we identified new potent VchCA inhibitors, some of them displayed high affinity toward VchCAγ class, for which few inhibitors are currently reported in literature. The best interesting VchCAγ inhibitor (*S*)‐N‐(1‐oxo‐1‐((4‐sulfamoylbenzyl)amino)propan‐2‐yl)furan‐2‐carboxamide (**40**) resulted more active and selective inhibitor when compared with acetazolamide (AAZ) as well as previously reported VchCA inhibitors.

## Graphical abstract

**Design in the time of cholera**: Carbonic anhydrases (CAs) catalyze the reversible hydration of CO_2_ in *Vibrio cholerae*, so VchCAs are attractive targets for the treatment of cholera. A structure‐inspired drug design of benzenesulfonamides led to identification of potent and selective VChCAα and VChCAγ inhibitors as potential antimicrobial agents. Further studies might furnish insight into the future design of effective agents able to fight antimicrobial resistance issue.

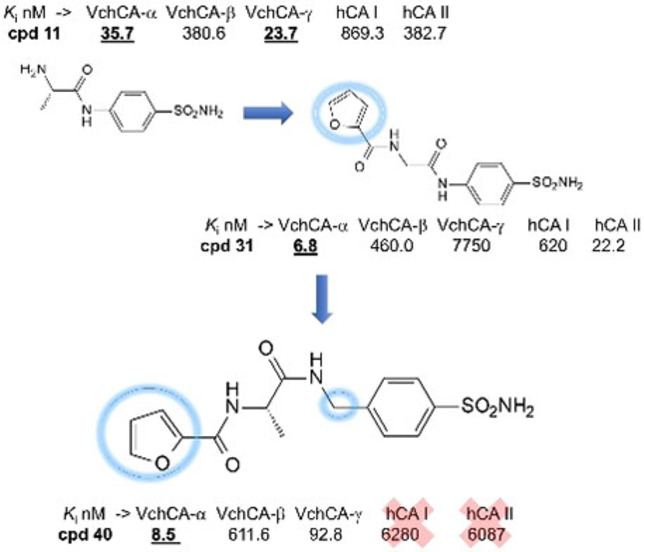

## Introduction

The challenge against bacterial diseases has been rekindled as various bacteria developed resistance to clinically used therapeutics. It is estimated that antimicrobial resistance (AMR) is responsible for ≅25,000 deaths a year worldwide. Hence, an emerging goal is to fight bacterial diseases by using drugs that possess distinct mechanisms of action, entering the so‐called post‐antibiotic era. The Gram‐negative *Vibrio cholerae* (Vch) is the causative agent of severe diarrheal events due to endemic diseases in Southeast Asian, African, and South American countries.[^1^, ^2^] The protein ToxT is the transcription factor involved in the activation of Vch virulence gene expression; ToxT is negatively regulated by the presence of unsaturated fatty acids (UFAs) of the bile, whereas it is positively induced by the presence of bicarbonate ion (HCO_3_^−^) secreted as pH buffer by the epithelial cells of gastro‐intestinal lumen colonized by Vch.^[3]^ The increase of bicarbonate ion outside of the bacterial cells is controlled by carbonic anhydrases (CAs, EC 4.2.1.1), that are a metalloenzymes catalyzing the reversible hydration of carbon dioxide (CO_2_). The superfamily of CAs is divided in eight genetically distinct classes (α‐, β‐, γ‐, δ‐, ζ‐, η ‐, θ‐, and ι‐classes) differing in terms of structure and metal ion located in the catalytic site (zinc, iron, cobalt and cadmium) and are diffused in vertebrates, protozoa, algae and bacteria.[^4^, ^5^] Vch genome encodes CAs belonging to α‐, β‐, γ‐classes (VchCAα, VchCAβ, and VchCAγ) that might be considered amenable targets for the development of therapeutics to fight Vch colonization. The α‐ and β‐ CAs are Zn(II) metalloenzymes, whereas γ‐ CA classes are Fe(II) metalloenzymes, even if they are also active with Zn(II) or Co(II) metal ions in the catalytic site. The coordination of metal ions involves three His residues in the α‐ and γ‐classes, whereas one His and two Cys residues are present in the β‐class.[^5^, ^6^] The VchCAα is a catalytically active monomer and possesses all the common features as the other α‐CAs (e. g. human expressed enzymes), except for the absence of four amino acids loops that makes these proteins more compact and smaller.^[7]^ VchCAβ is a homotetramer for which the catalytic site is localized at the interface of one dimer in a closed conformation called *T‐state*, inactive at pH values below 8.0, and then converted to the open conformation when pH is greater than 8.0.^[6]^ Lastly, the γ‐class is a homotrimer and seems to adopt a “closed” conformation like the “*T‐state*” form of VchCAβ as shown for the γ‐class homologue Yrda from *E. Coli* (PDB code: 3TIS).^[8]^

**Figure 1:**
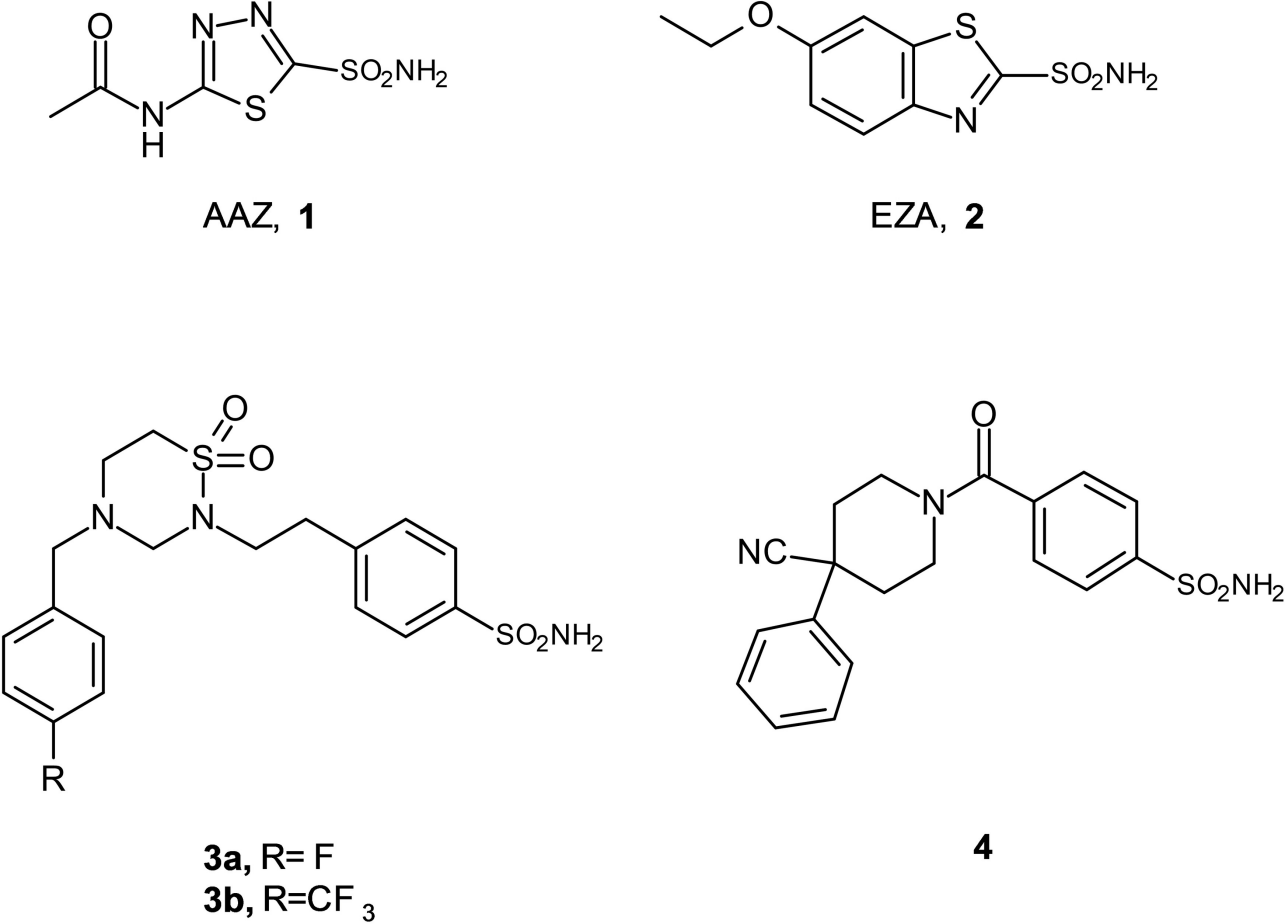
Chemical structure of well‐known VchCA inhibitors: acetazolamide (**AAZ**), ethoxzolamide (**EZA**), 4‐(2‐(4‐(4‐fluorobenzyl)‐1,1‐dioxido‐1,2,4‐thiadiazinan‐2‐yl)ethyl)benzenesulfonamide (**3 a**) 4‐(2‐(1,1‐dioxido‐4‐(4‐(trifluoromethyl)benzyl)‐1,2,4‐ thiadiazinan‐2yl)ethyl)benzenesulfonamide (**3 b**) ^[12]^ and 4‐(4‐cyano‐4‐phenylpiperidine‐1‐carbonyl)benzenesulfonamide (**4**) ^[22]^.

Several sulfonamides demonstrated the ability to affect the activity of CAs from distinct bacterial species. These compounds possess a crucial moiety able to act as zinc binder group (ZBG) within catalytic site of CAs. From the various studies, Acetazolamide (AAZ, **1**) and ethoxzolamide (EZA, **2**) (see Figure 1) demonstrated good inhibitory profile toward VchCAs[^5^, ^9^] and EZA suppressed bicarbonate mediated virulence gene induction, thus reducing the growth rates of pathogen.[^5^, ^10^, ^11^] In the last years, many scientific efforts have been addressed to develop of VchCA inhibitors with the aim to identify new candidates for the therapeutic treatment of cholera disease in humans. The main criterion to consider VchCA inhibitors as valuable antimicrobial agents is their ability to selectively target the bacterial CAs over the human expressed homologue enzymes. Among the reported antimicrobial CAIs, the 1,2,4‐thiadiazinane‐1,1‐dioxide derivatives **3 a** and **3 b** have emerged as exciting agents, that showed good VchCA selectivity over the physiologically dominant human isoenzymes.^[12]^

Looking for new potential application of CAIs from the in‐house library of sulfonamides synthesized in our laboratory,[^13^, ^14^, ^15^, ^16^, ^17^, ^18^, ^19^, ^20^, ^21^] we have selected a small set of compounds that resulted to strongly affect the carbon dioxide hydrase activity of VchCAα.^[22]^ These compounds moderately acted against the β‐class, but none of these proved to be capable of influencing the VchCAγ activity. Among this series of compounds, the best outcome was obtained for the 4‐(4‐cyano‐4‐phenyl‐piperidine‐1‐carbonyl)benzenesulfonamide (**4**), which combined nanomolar activity against VchCAα (*K*_i_=89.9 nM) with sub‐micromolar affinity against VchCAβ (*K*_i_=806.4 nM).^[22]^

**Figure 2:**
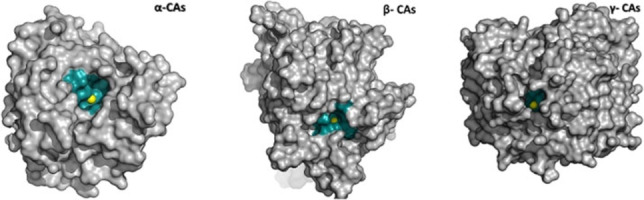
3D‐structures of bacterial α‐, β‐, γ‐CAs with focus on catalytic pocket size. The figure was generated with PyMOL (https://pymol.org) by using structural data retrieved from 5TUO, 1KEJ, and 3TIS accession codes in protein databank archive (https://www.rcsb.org)

From the structural data collected for these three classes of bacterial CAs, we might observe that VchCAβ and VchCAγ possess a narrower active site (Figure 2) when compared with VchCAα;^[23]^ therefore, the different structural organization might explain the different degree of affinity/inhibition measured for several studied compounds bearing the sulfonamide pharmacophore linked to bulky groups.

Based on our knowledge, we now reported the design and synthesis of two novel sets of 2‐(hetero)arylformamido‐N‐(4‐sulfamoylphenyl)alkylamides and (hetero)arylformamido‐N‐[(4‐sulfamoylphenyl]methyl)alkylamides. These compounds were biological screened by a stopped‐flow carbon dioxide assay to investigate their ability to affect the hydrase activity of VchCAs, thus furnishing considerable SAR information for future compounds optimization.

## Results and Discussion

### Design and synthesis

**Figure 3:**
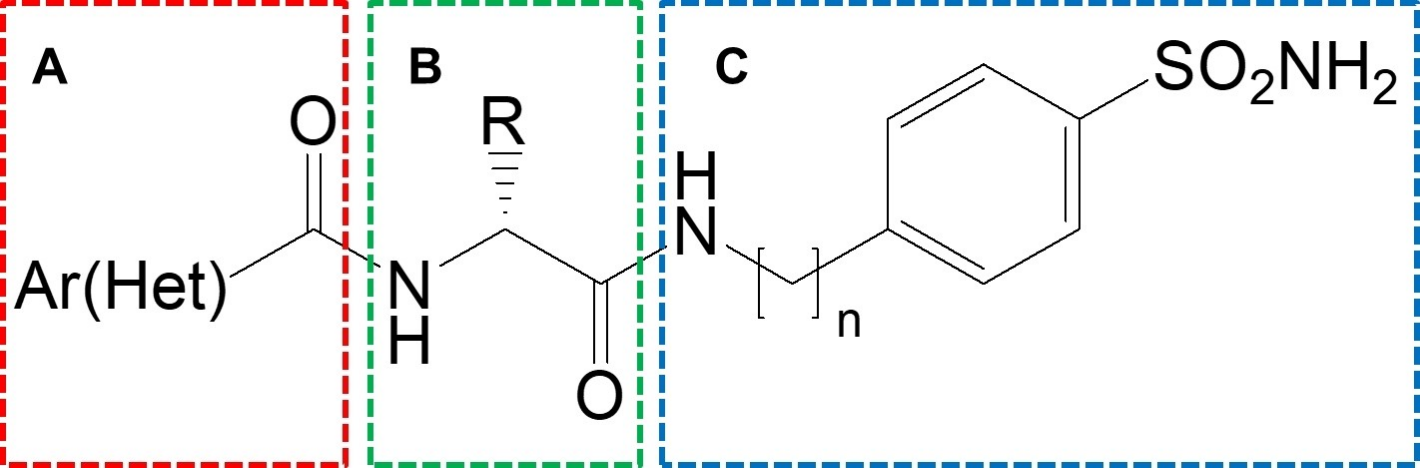
Schematic representation of designed sulfonamides.

This paper presents a series of benzenesulfonamide derivatives in which the cycloalkylamine nucleus of the previously reported VchCA inhibitors (e. g. compound **4**) was replaced by more flexible and less bulky amino acid linking groups as depicted in Figure 3. The bivalent chemical properties of the amino acids make them optimal candidates to link together two different molecular portions, generating compounds with improved characteristic such as good pharmacokinetic properties. Again, the linker moiety could efficiently establish ancillary interactions with hydrophobic/hydrophilic walls of VchCA cavity thus controlling the enzymatic affinity.

In the first subset of designed compounds we focused our attention on the modification of the Ar(Het) cap group in combination with a variation of the side chain ‐R1 of the amino acid spacer. Whereas, in the second subset we investigated how the homologation of the sulfanilamide portion might influence the enzyme binding orientation within VchCAs, thus affecting the affinity. The two subsets of 2‐(hetero)arylformamido‐N‐(4‐sulfamoylphenyl)alkylamides and (hetero)arylformamido‐N‐[(4‐sulfamoylphenyl]methyl)alkylamides were synthesized by means of a multi‐step process. All new synthesized compounds were screened for their potential CA inhibitory property against VchCAα, VchCAβ, and VchCAγ and Ki values were compared to the off‐target human CA I and CA II, thus furnishing suggestions for further development of anticholera agents affecting CA activity.

### Chemistry

**Scheme 1:**
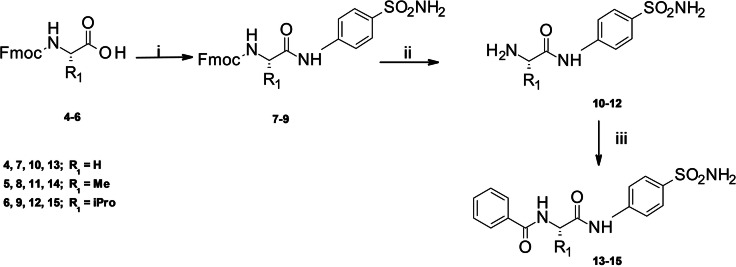
Reagents and conditions: i) *p*‐aminobenzenesulfonamide, HATU, DIPEA, DMF, overnight, 0 °C to rt; ii) DBU, DMF, 20 min, rt.; iii) PhCOCl, DMF/DCM, 1 h, rt.

**Scheme 2:**
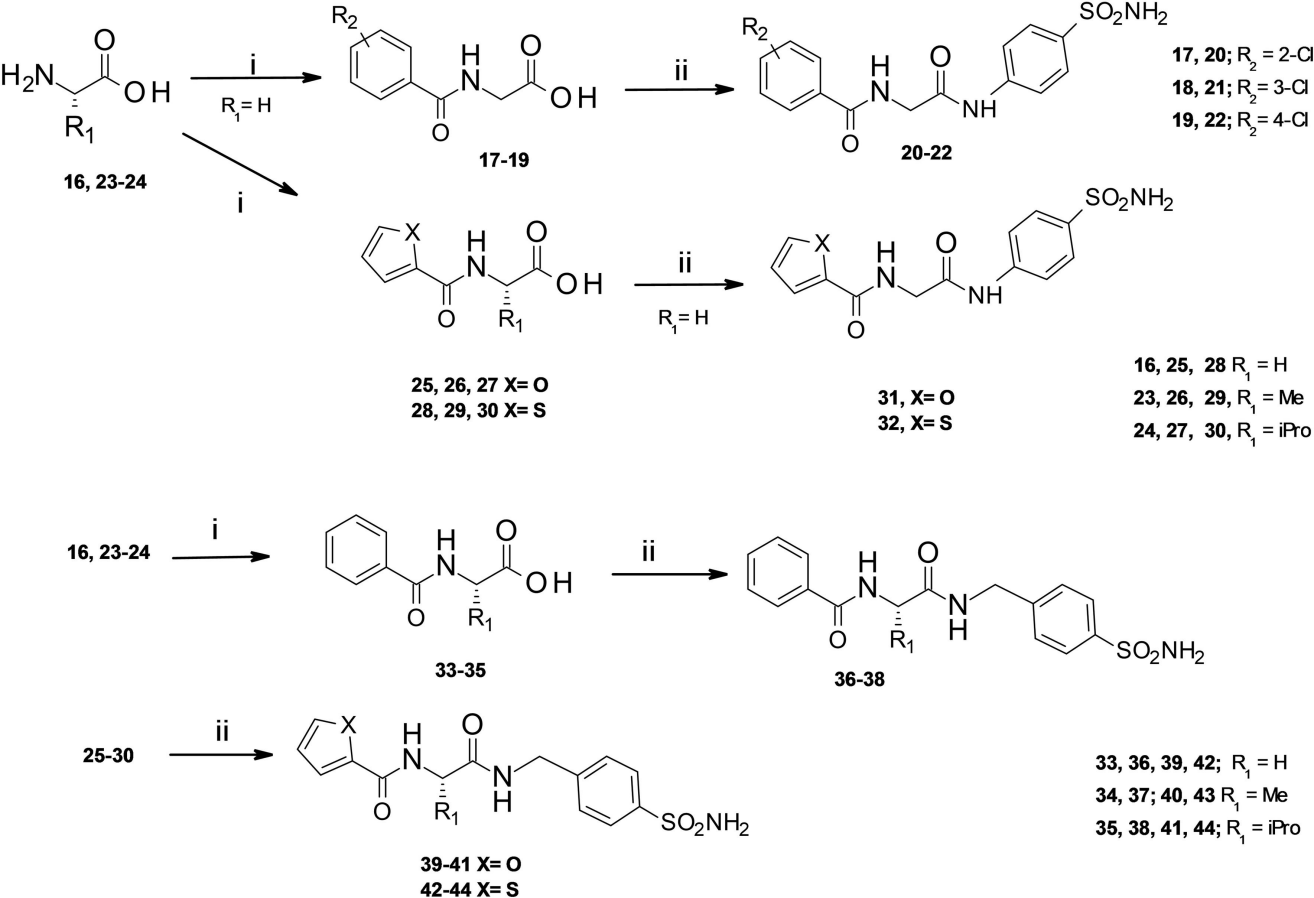
Reagents and conditions: i) (hetero)arylCOCl, NaOH, H_2_O/MeCN (v/v 2 : 1), 2.5 h, from 0 °C to rt; ii) p‐aminobenzenesulfonamide or 4‐(aminomethyl)benzenesulfonamide, HBTU, DIPEA, DMF, overnight, rt.

The synthetic route of designed 2‐(hetero)arylformamido‐N‐(4‐sulfamoylphenyl)alkylamides (**10**–**15**, **20**–**22**, **31**–**32**) and (hetero)arylformamido‐N‐[(4‐sulfamoylphenyl]methyl)alkylamides (**36**–**44**) is illustrated in Schemes 1–2. First, the Fmoc‐L‐amino acids **4**–**6** were coupled with p‐aminobenzenesulfonamide to afford the key intermediates **7**–**9** (Scheme 1); the preparation of amides **7**–**9** was performed by using different coupling reagents as (2‐(1H‐benzotriazol‐1‐yl)‐1,1,3,3‐tetramethyluronium hexafluorophosphate (HBTU), (1‐[bis(dimethylamino)methylene]‐1H‐1,2,3‐triazolo[4,5‐b]pyridinium‐3‐oxide‐hexafluorophosphate) (HATU) and (1‐cyano‐2‐ethoxy‐2‐oxoethylidenaminooxy)dimethylaminomorpholinocarbenium hexafluorophosphate) (COMU). The best result was obtained by using HATU in ice bath, thus minimizing the formation of side products. Then the intermediates **7**–**9** were deprotected by using 1,8‐diazabiciclo[5.4.0]undec‐7‐ene (DBU) to afford compounds **10**–**12**. Thereafter, the target compounds **13**–**15** were obtained by reaction with benzoyl chloride.

Secondly, we selected the free amino acids **16**, **23**–**24** as useful synthons to reduce the reaction steps. Indeed, the key intermediates N‐(hetero)aroyl‐amino acids **17**–**19, 25**–**30** and **33**–**35** were readily obtained from the commercially available L‐amino acids **16**, **23**–**24** and the appropriate (hetero)aroyl chloride in alkaline medium (Scheme 2). Finally, the intermediates **17**–**19, 25**–**30** and **33**–**35** were treated with the activating agent HBTU and coupled with *p*‐aminobenzenesulfonamide or 4‐(aminomethyl)benzenesulfonamide to afford the final compounds **20**–**22** and **31**–**32** as well as the homologous set of compounds **36**–**44**, respectively (Scheme 2).

It is worth to stress that both the amino acids and Fmoc‐L‐protected amino acids rapidly reacted in the mild reaction conditions thus generating low probability of chiral inversion. The structure of all the final compounds was confirmed on the basis of their ^1^H‐NMR and ^13^C‐NMR spectral data and supported by elemental analysis.

### Carbonic anhydrase inhibition assay

The inhibitory effects of the designed 2‐(hetero)arylformamido‐N‐(4‐sulfamoylphenyl)alkylamides **13**–**15**, **20**–**22**, **31**–**32** and 2‐(hetero)arylformamido‐N‐[(4‐sulfamoylphenyl]methyl)alkylamides **36**–**44** were measured against VchCAα, ‐β and ‐γ by means of a stopped‐flow carbon dioxide hydrase assay.^[24]^ In the Table 1 we collected the obtained Ki values for all tested compounds in comparison with amine **10**–**12** as well as **1**, **3 a**, **3 b**, and **4**, which we used as reference compounds. Data for the physiologically ubiquitous human isoforms CA I and II are included for comparison and to analyze the selectivity profile of tested compounds.

**Table 1:** Inhibition data of VchCA‐α, ‐β and ‐γ for benzenesulfonamide derivatives **13**–**15**, **20**–**22**, **31**–**32**, **36**–**44** in comparison with **3 a**, **3 b**, **4** and **AAZ (1)** as reference compounds.

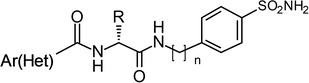								
				*K*_i_ [nM]^[a]^				
	Ar(Het)	R	n	VchCA α	VchCA β	VchCA γ	hCA I	hCA II
**10**	–	H	0	8.0	3169	743.8	390.0	50.2
**11**	–	Me	0	35.7	380.6	23.7	869.3	382.7
**12**	–	iPro	0	658.8	362.5	88.6	608.7	182.6
**13**	C_6_H_5_	H	0	0.4	884	562.8	524.3	19.9
**14**	C_6_H_5_	Me	0	9.5	3000	80.5	46.1	4.4
**15**	C_6_H_5_	iPro	0	9.2	389.3	24.3	51.5	3.7
**20**	2‐ClC_6_H_4_	H	0	8.9	795.5	8933	564.1	29.6
**21**	3‐ClC_6_H_4_	H	0	18.5	82.8	363.6	127.2	14.7
**22**	4‐ClC_6_H_4_	H	0	9.6	59.7	282.2	81.3	3.3
**31**	Furan‐2‐yl	H	0	6.8	460.0	7750	620.8	22.2
**32**	Thiophen‐2‐yl	H	0	4.8	635.7	6025	429.6	38.5
**36**	C_6_H_5_	H	1	8.2	82.8	230.1	753.8	367.6
**37**	C_6_H_5_	Me	1	56.5	93.3	261.0	722.2	301.7
**38**	C_6_H_5_	iPro	1	37.6	94.6	331.6	504.8	169.8
**39**	Furan‐2‐yl	H	1	35.7	393.5	733.9	1435	315.6
**40**	Furan‐2‐yl	Me	1	8.5	611.6	92.8	6280	6087
**41**	Furan‐2‐yl	iPro	1	97.8	707.0	95.3	215.4	19.6
**42**	Thiophen‐2‐yl	H	1	9.3	626.8	244.5	828.3	87.3
**43**	Thiophen‐2‐yl	Me	1	63.1	690.8	93.2	650.5	71.1
**44**	Thiophen‐2‐yl	iPro	1	30.5	94.4	84.8	94.9	18.4
**3 a** ^[b]^	–	–	‐	6.1	210.9	8235	1905	86.7
**3 b** ^[b]^	–	–	‐	810.7	40.6	8666	647.8	37.2
**4** ^[c]^	–	–		89.9	806.4	>10.000	6.5	0.6
**AAZ** ^[b]^	–	–		6.8	451	473	250	12.1

[a] Errors in the range of ±10 % of the reported value, from three different assays. [b] Data taken from reference [12]. [c] Data taken from reference [13].

The biochemical data highlighted the ability of almost all investigated new compounds to effectively affect the CO_2_ hydrase activity of the three VchCA classes, thus confirming the proof‐of‐concept that flexible ligands might be efficient inhibitors. Interestingly, the new benzenesulfonamides generally displayed inhibitory effects toward VchCAγ higher than those of reference compounds **3 a**, **3 b** and **4**. All tested benzenesulfonamides were more potent inhibitors of hCA II when compared to hCA I isoform.

In more detail, the following SAR toward the three classes of VhCAs can be drawn out of the data reported in Table 1:

- VchCAα is strongly inhibited by all the studied compounds that demonstrated K_i_ values spanning from 0.4 to 97.8 nM. Among the first series of compounds containing the 4‐aminobenzenesulfonamide moiety (n=0), the most active inhibitor resulted the unsubstituted molecule **13** (R_1_=H, K_i_=0.4 nM). The branching modification by introduction of methyl or isopropyl group on 2‐formylacetamide linker reduced the activity as found for compounds **14**–**15**. In a similar way, the presence of chlorine atom on phenyl ring (i. e. compounds **20**–**22**) as well as the introduction of furyl (for compound **31**) or thiophenyl ring (for compound **32**), resulted in a moderate decline of the inhibitory potency thus providing ligands with a slight reduction of affinity (K_i_ values in the range of 4.8–18.5 nM). Concerning the second series of tested compounds bearing the 4‐aminomethylbenzenesulfonamide as zinc binding moiety, no significant improvement in VchCAα affinity was observed when compared to the first series of parent compounds **13**–**15** and **31**–**32**. Interestingly, the homologation has generally led to a promising selectivity over human α‐CAs hCA I/II, especially for compound **40** that was about‐730‐fold more active against VchCAα over hCA I.
- VchCAβ was moderately to poorly inhibited by this novel class of sulfonamides, that demonstrated K_i_ values spanning from 59.7 to 3000 nM. The best active inhibitors **21, 22, 36, 37, 38** and **44** belong both to the series of aminobenzenesulfonamides and methylaminobenzenesulfonamides. No clear SAR correlation emerged from the analysis of the K_i_ data for tested compounds containing the performed structural tail modifications. However, for compounds **36**–**38** (n=1) we found higher inhibitory potency when compared to corresponding analogs **13**–**15** (n=0) for which the benzenesulfonamide moiety is directly connected to nitrogen atom of acetamide linker.
- The hydrase activity of VchCAγ was affected by all tested compounds with the best result obtained for aminobenzenesulfonamide inhibitor **15** (K_i_=24.3 nM) for which we measured inhibitory effects higher than that of reference compound AAZ (K_i_=473 nM). Unfortunately, the outlier molecule **15** demonstrated low selectivity over hCA I and hCA II isoforms. On the contrary, the mild active compound **40** (K_i_=92.8 nM toward VchCAγ) proved to be ∼70‐fold more selective over hCA I and hCAII (K_i_=6280 nM and K_i_=6097 nM, respectively)

Collecting these results, it emerged that trivial changes into the linker seems to strongly influence the inhibition potency/selectivity against the three classes of VchCAs. The most considerable changes in activity have been observed especially for the homologous subset of compounds namely methylaminobenzenesulfonamides **36**–**44**, thus suggesting that a simple variation in chain length can affect the spatial relationship of functional groups in molecule thereby influencing the enzyme binding recognition.

## Conclusion

We have developed a new series of twenty benzenesulfonamides bearing amino acids as linking groups by applying a structural simplification approach. Our studies have allowed us to identify several potent inhibitors of VchCAs, that might be useful to provide new agents able to fight symptoms associated with a cholera infection. From kinetic studies, we revealed that several sulfonamides (e. g. **15**, **36**, **37** and **40**) showed the ability to preferentially inhibit the microbial CAs over human isozymes. This study highlighted that the presence of a flexible linker is an optimal chemical feature to design efficacious VchCAIs, thus providing relevant SAR information that may be exploited for the design of novel potential anti‐infective agents. Considering the high level of sequence homology found for VchCAs to further CAs from other pathogen bacteria, it is possible to speculate that the newly identified VchCAIs could be employed as a template in designing selective sulfonamides targeting α, β and γ CA classes expressed in other human pathogens.

## Experimental Section

### Chemistry

All reagents were obtained from common commercial suppliers and were used without further purification. For flash column chromatography a CombiFlash® Rf+ from Teledyne ISCO was used. Melting points were determined on a Buchi B‐545 apparatus (BUCHI Labortechnik AG Flawil, Switzerland) and are uncorrected. By combustion analysis (C, H, N) (Carlo Erba Model 1106‐Elemental Analyzer) we determined the purity of synthesized compounds; the results confirmed a ≥95 % purity. Thin‐layer chromatography was performed on aluminum sheets from Merk (Silica gel 60 F254, 20×20). Chromatograms were visualized by UV light (λ=254 nm/ 366 nm) or by staining with ninhydrin solution. ^1^H‐NMR and ^13^C‐NMR spectra were measured in dimethylsulfoxide‐d_6_ (DMSO‐d_6_) or Deuterochloroform (CDCl_3_‐d) with a Varian Gemini 500 spectrometer (Varian Inc. Palo Alto, California USA) or with a Bruker Avance III™ HD 400 MHz NMR system equipped with Prodigy cryo‐probe. Chemical shifts are quoted in δ (ppm) and coupling constants (*J*) in Hertz. All exchangeable protons were confirmed by addition of D_2_O. High‐resolution mass spectra were recorded on an Agilent 5975 C MSD Quadrupol, Q‐Tof micro from Waters or LTQ Orbitrap XL from Thermo Fisher Scientific. HPLC‐MS measurements were performed on an LCMS‐2020 system from Shimadzu equipped with a Luna® C18(2) column (3 μm, 100 A, 100×4.6 mm) using a linear gradient of CH_3_CN+0.05 % HCOOH (5→95 % in 9 min) in H_2_O+0.05 % HCOOH at a flow rate of 1.0 mL/min.

### General procedures for the synthesis of (9H‐fluoren‐9‐yl)methyl(2‐oxo‐2‐((4‐sulfamoylphenyl)amino)ethyl)carbamate derivatives (7–9)

To a solution of N‐α‐Fmoc‐protected amino acids (1 molar equivalent, **4**–**6**) in dry dimethylformamide (DMF) (2 mL), 1‐[Bis(dimethylamino)methylene]‐1H‐1,2,3‐triazolo[4,5‐b]pyridinium‐3‐oxid‐hexafluorophosphate (HATU) (1 molar equivalent) was added at 0 °C and the mixture was stirred vigorously at 450 rpm. Then, N,N‐Diisopropylethylamine (DIPEA) (2.5 molar equivalents) and p‐aminobenzenesulfonamide (1.25 molar equivalent) were added. The reaction mixture was vigorously stirred at room temperature overnight. Water (10 mL) was added and the mixture was extracted with EtOAc (3×10 mL). The organic phase was washed with acidic water (pH=4–5), dried with Na_2_SO_4_ and concentrated until dryness under reduced pressure. The crude was sonicated with Et_2_O and filtered to give the desired intermediates **7**–**9** as white powder.

**(9H‐Fluoren‐9‐yl)methyl(2‐oxo‐2‐(4‐sulfamoylphenyl)amino)ethyl‐carbamate (7)** Yield: 79 %; m.p.: 224–225 °C; t_R_: 5.41’ ^1^H‐NMR (400 MHz, DMSO‐d_6_): (δ) 3.84 (m, 2H, CH_2_), 4.26 (m, 1H, CH), 4.32 (m, 2H, CH_2_), 7.25 (bs, 2H, NH_2_), 7.33–7.43 (m, 4H, ArH), 7.68–7.76 (m, 7H, ArH, NH), 7.90–7.92 (m, 2H, ArH), 10.32 (s, 1H, NH); ^13^C‐NMR (126 MHz, DMSO‐d_6_): 44.5, 47.1, 66.2, 119.0, 120.6, 125.7, 127.2, 127.5, 128.1, 138.8, 141.2, 142.2, 144.3, 157.1, 169.0. Anal. for C_23_H_21_N_3_O_5_S: C, 61.19; H, 4.69; N, 9.31. Found: C, 61.21 %; H, 4.73 %; N, 9.27 %. LRMS (ESI): m/z Calcd. for [MH]^+^ 451.12. Found: 452.00.

**(S)‐(9H‐Fluoren‐9‐yl)methyl(1‐oxo‐1‐(4‐sulfamoylphenyl)amino)propan‐2‐ylcarbamate (8)** Yield: 58 %; m.p.: 229–230 °C; t_R_: 5.52’; ^1^H‐NMR (400 MHz, DMSO‐d_6_): (δ) 1.33 (m, 3H, CH_3_), 4.20–4.31 (m, 4H, CH_2_, CH), 7.26 (bs, 2H, NH_2_), 7.32‐7.45 (m, 4H, ArH), 7.73–7.77 (m, 7H, ArH, NH), 7.86–7.91 (m, 2H, ArH), 10.34 (s, 1H, NH); ^13^C‐NMR (126 MHz, DMSO‐d_6_): 18.3, 47.1, 51.3, 66.1, 119.2, 120.6, 125.7, 127.1, 127.5, 128.1, 138.8, 141.2, 144.2, 144.3, 156.3, 172.7. Anal. for C_24_H_23_N_3_O_5_S: C, 61.92; H, 4.98; N, 9.03. Found: C, 62.05 %; H, 4.85 %; N, 9.09 %. LRMS (ESI): m/z Calcd. for [MH]^+^ 465.14. Found: 466.00.

**(S)‐(9H‐Fluoren‐9‐yl)methyl(3‐methyl‐1‐oxo‐1‐(4‐sulfamoylphenyl)amino)butan‐2‐ylcarbamate (9)** Yield: 73 %; m.p.: 230–231 °C; t_R_: 5.90’; ^1^H‐NMR (400 MHz, DMSO‐d_6_): (δ) 0.91–0.95 (m, 6H, CH_3_), 4.01–4.10 (m, 2H, CH), 4.22–4.30 (m, 3H, CH_2_, CH), 7.25 (bs, 2H, NH_2_), 7.31–7.43 (m, 4H, ArH), 7.74–7.76 (m, 7H, ArH, NH), 7.88–7.90 (m, 2H, ArH), 10.41 (s, 1H, NH). Anal. for C_26_H_27_N_3_O_5_S: C, 63.27; H, 5.51; N, 8.51. Found: C, 63.39 %; H, 5.28 %; N, 8.63 %. LRMS (ESI): m/z Calcd. for [MH]^+^ 493.17. Found: 494.00.

### General procedures for the synthesis of amines 10–12

The N‐α‐Fmoc‐protected intermediates **7**–**9** (250 mg) were treated with 2 % (v/v) of 1,8‐Diazabicyclo[5.4.0]undec‐7‐ene (DBU) in dimethylformamide (DMF, 2.5 mL). The reaction mixture was stirred at room temperature for 20 minutes. After the starting material has disappeared, the crude product was concentrated in vacuo and then subjected to sonication with a n‐hexane (Hex)‐dichloromethane (DCM) solution (50 %, v/v) to provide the free amines **10**–**12** as white powder. The registered CAS numbers for resulting compounds **10**–**12** have been already assigned as reported below The experimental properties for 2‐amino‐N‐(4‐sulfamoylphenyl)acetamide, (compound **10** CAS Number: 133639–71‐1) matched with those reported in literature.^[25]^ For compounds **11** and **12** no structural data are reported in literature.

**(S)‐2‐Amino‐N‐(4‐sulfamoylphenyl)propanamide (11) (CAS number 1163296‐78‐3)** Yield: 68 %; m.p.: 216–217 °C; tR: 0.43’; 1H‐NMR (400 MHz, CD3OD‐d4): (δ) 1.37 (m, 3H, CH3), 3.58 (m, 1H, CH), 7.77–7.87 (m, 4H, ArH). 13 C‐NMR (126 MHz, DMSO‐d6): 21.5, 51.4, 118.9, 126.8, 138.4, 142.1, 175.8. Anal. for C_9_H_13_N_3_O_3_S: C, 44.43 %; H, 5.39 %; N, 17.27 %. Found: C, 44.51 %; H, 5.27 %; N, 17.31 %. HRMS (ESI): m/z Calcd. for [MH]+ 243.28. Found: 244.00.

**(S)‐2‐amino‐3‐methyl‐N‐(4‐sulfamoylphenyl)butanamide (12) (CAS number 96313‐17‐6)** Yield: 62 %; m.p.: 209–210 °C; tR: 0.84’; 1H‐NMR (400 MHz, DMSO‐d6): (δ) 0.83–0.92 (m, 6H, CH3), 1.88‐1.96 (m, 2H, CH), 7.23 (bs, 2H, NH2), 7.73–7.81 (m, 4H, ArH). Anal. for C_11_H_17_N_3_O_3_S: C, 48.69 %; H, 6.32 %; N, 15.49 %. Found: C, 48.81 %; H, 6.26 %; N, 15.52 %. LRMS (ESI): m/z Calcd. for [MH]+ 271.34. Found: 272.00.

### General procedures for the synthesis of target benzamides 13–15

To a stirred solution of amines **10**–**12** (1 molar equivalent) in dimethylformamide (DMF)/dichloromethane (DCM) (50 %, v/v), benzoyl chloride (0.8 molar equivalent) was added in ice bath. The resulted mixture was stirred at room temperature for 1 hour. After the disappearance of the starting material, the organic solvent was evaporated in vacuo and purified by flash chromatography (eluting with 0–20 % v/v of MeOH in DCM). The final crude was sonicated with petroleum ether (PE)/ethanol (EtOH) (50 %, v/v) to afford the desired compounds **13**–**15** as white powder.

**(S)‐N‐(2‐Oxo‐2‐((4‐sulfamoylphenyl)amino)ethyl)benzamide (13)** Yield: 36 %; m.p.: 214–216 °C; ^1^H‐NMR (500 MHz, DMSO‐d_6_): (δ) 4.08 (d, J=5.96 Hz, 2H, CH_2_), 7.23 (bs, 2H, NH_2_), 7.47–7.55 (m, 3H, ArH), 7.75 (m, 4H, ArH), 7.89 (m, 2H, ArH), 8.86 (t, J=5.96 Hz, 1H, NH), 10.39 (s, 1H, NH); ^13^C‐NMR (126 MHz, DMSO‐d_6_): (δ) 43.8, 119.1, 120.3, 127.2, 127.8, 128.8, 131.9, 132.4, 134.3, 138.8, 142.6, 167.1, 168.8. Anal. for C_15_H_15_N_3_O_4_S: C, 54.04 %; H, 4.54 %; N, 12.61 %; Found: C, 54.12 %; H, 4.62 %; N, 12.70 %.

**(S)‐N‐(1‐oxo‐1‐((4‐sulfamoylphenyl)amino)propan‐2‐yl)benzamide (14)** Yield: 54 %; m.p.: 260–261 °C; t_R_: 4.09’; ^1^H‐NMR (400 MHz, DMSO‐d_6_): (δ) 1.96‐1.98 (m, 3H, CH_3_), 5.20–5.23 (m, 1H, CH), 6.89–6.90 (m, 1H, NH), 7.87–8.00 (m, 3H, ArH), 8.23–8.28 (m, 4H, ArH), 8.39–8.48 (m, 2H, ArH), 10.23 (bs, 1H, NH), ^13^C‐NMR (126 MHz, DMSO‐d_6_): (δ) 17.6, 50.3, 118.9, 127.1, 128.0, 128.6, 131.8, 134.3, 138.7, 142.5, 166.8, 172.6. Anal. for C_16_H_17_N_3_O_4_S: C, 55.32 %; H, 4.93 %; N, 12.10 %. Found: C, 55.47 %; H, 4.79 %; N, 12.21 %. HRMS (ESI): m/z Calcd. for [MH]^+^ 347.09. Found: 348.00.

**(S)‐N‐(3‐methyl‐1‐oxo‐1‐((4‐sulfamoylphenyl)amino)butan‐2‐yl)benzamide (15)** Yield: 63 %; m.p.: 279–280 °C; t_R_: 4.09’; ^1^H‐NMR (400 MHz, DMSO‐d_6_): (δ) 0.97–1.02 (m, 6H, CH_3_), 2.20‐2.25 (m, 1H, CH), 4.39–4.45 (t, J=8.2 Hz, 1H, CH), 7.23 (bs, 2H, NH_2_), 7.46–7.57 (m, 3H, ArH), 7.75–7.81 (m, 4H, ArH), 7.90–7.93 (m, 2H, ArH), 8.55–8.57 (d, J=8.2 Hz, 1H, NH), 10.54 (bs, 1H, NH). Anal. for C_18_H_21_N_3_O_4_S: C, 57.58 %; H, 5.64 %; N, 11.19 %‐ Found: C, 57.62 %; H, 5.58 %; N, 11.26 %. HRMS (ESI): m/z Calcd. for C_18_H_21_N_3_O_4_S, [MH]^+^ 376.13. Found: 376.00.

### General synthetic procedures for (hetero)aroylamino acid derivatives 17–19, 25–30 and 33–35

The appropriate L‐amino acid derivatives **16**, **23** or **24** (1 molar equivalent) and NaOH pellets (3 molar equivalents), were dissolved in H_2_O/CH_3_CN (2 : 1 v/v, 6 mL) and the resulting mixture was cooled at 0° C. Then, the appropriate aroyl chloride (0.8 molar equivalent) was added in three portions within 1 hour by stirring vigorously at 450 rpm. The solution was brought to room temperature and kept under stirring for another 1.5 hour. Later, concentrated hydrochloric acid (37 % w/w, HCl) was added slowly to the mixture with continuous stirring until it became acidic (pH=3), which resulted into the precipitation of crude product, which was collected by vacuum filtration, washed with cold Et_2_O and dried in vacuo at room temperature to give the desired known intermediates **17**–**19, 25**–**30** and **33**–**35**. The registered CAS numbers for compounds **17–19, 25‐30 and 33–35** have been already assigned as reported in Table 2. The experimental properties **for 17–19, 25–30** and **33–35** matched with those reported in literature.[^26^, ^27^, ^28^, ^29^, ^30^, ^31^, ^32^]

**Table 2:** Registered CAS numbers for compounds **17**–**19**, **25**–**30** and **33**–**35**.

entry	CAS number	entry	CAS number
**17**	16555‐60‐5	**28**	33955‐17‐8
**18**	57728‐59‐3	**29**	2163783‐67‐1
**19**	13450‐77‐6	**30**	854007‐21‐9
**25**	5657‐19‐2	**33**	495‐69‐2
**26**	1001616‐79‐0	**34**	1205‐02‐3
**27**	1361143‐20‐5	**35**	2901‐80‐6

### General synthetic procedures for the 2‐(hetero)arylformamido‐N‐(4‐sulfamoylphenyl)alkylamide derivatives (20–22 and 31–32)

To a solution of the appropriate aroyl glycine derivatives (**17**–**19, 25** and **28**) in N,N‐dimethylformamide (DMF) (2 mL), N,N,N’,N’‐tetramethyl‐O‐(1H‐benzotriazol‐1‐yl)‐uraniumhexafluorophosphate (HBTU (1 molar equivalent) was added. The mixture was stirred at room temperature for 15 minutes. Then, 1,8‐Diazabicyclo[5.4.0]undec‐7‐ene (DBU) (2.5 molar equivalents) and p‐aminobenzenesulfonamide (1 molar equivalent) were added. The reaction mixture was left overnight at room temperature and then quenched with H_2_O (10 mL) and extracted with EtOAc (3×10 mL). The organic phase was washed with saturated NaCl solution, dried with Na2SO4 and concentrated until dryness under reduced pressure. The crude was purified by crystallization from EtOH to give the desired final compounds **20–22** and **31–32** as white powder.

**2‐Chloro‐N‐(2‐oxo‐2‐((4‐sulfamoylphenyl)amino)ethyl)benzamide (20)** Yield: 27 %; m.p.: 249–251 °C; 1H‐NMR (500 MHz, DMSO‐d6): (δ) 4.08 (d, J=5.87 Hz, 2H, CH2), 7.26 (bs, 2H, NH2), 7.42–7.53 (m, 4H, ArH), 7.77 (m, 4H, ArH), 8.77 (t, J=5.87 Hz, 1H, NH), 10.49 (s, 1H, NH); Anal. for C_15_H_14_ClN_3_O_4_S: C, 48.98 %; H, 3.84 %; N, 11.42 %; Found: C, 48.90 %; H, 3.76 %; N, 11.38 %.

**3‐Chloro‐N‐(2‐oxo‐2‐((4‐sulfamoylphenyl)amino)ethyl)benzamide (21)** Yield: 19 %; m.p.: 262–264 °C; 1H‐NMR (500 MHz, DMSO‐d6): (δ) 4.06 (d, J=5.87 Hz, 2H, CH2), 7.22 (bs, 2H, NH2), 7.31 (m, 2H, ArH), 7.74 (m, 4H, ArH), 7.96 (m, 2H, ArH), 8.66 (t, J=5.96 Hz, 1H, NH), 10.55 (s, 1H, NH); Anal. for C_15_H_14_ClN_3_O_4_S: C, 48.98 %; H, 3.84 %; N, 11.42 %; Found: C, 48.88 %; H, 3.74 %; N, 11.32 %;

**4‐Chloro‐N‐(2‐oxo‐2‐((4‐sulfamoylphenyl)amino)ethyl)benzamide (22)** Yield: 41 %; m.p. 226 – 228 °C; 1H‐NMR (500 MHz, DMSO‐d6): (δ) 4.07 (d, J=5.87 Hz, 2H, CH2), 7.23 (bs, 2H, NH2), 7.51–7.93 (m, 8H, ArH), 9.01 (t, J=5.87 Hz, 1H, NH), 10.39 (s, 1H, NH); Anal. for C_15_H_14_ClN_3_O_4_S: C, 48.98 %; H, 3.84 %; N, 11.42 %; Found: C, 48.78 %; H, 3.64 %; N, 11.22 %;

**N‐(2‐Oxo‐2‐((4‐sulfamoylphenyl)amino)ethyl)furan‐2‐carboxamide (31)** Yield: 24 %; m.p.: 238–240 °C; 1H‐NMR (500 MHz, DMSO‐d6): (δ) 4.03 (d, J=5.96 Hz, 2H, CH2), 6.62 (m, 1H, ArH), 7.14 (m, 1H, ArH), 7.23 (bs, 2H, NH2), 7.74 (bs, 4H, ArH), 7.85 (m, 1H, ArH), 8.66 (t, J=5.96 Hz, 1H, NH), 10.55 (s, 1H, NH); 13 C‐NMR (126 MHz, DMSO‐d6): (δ) 43.1, 112.3, 114.2, 119.0, 127.1, 138.7, 142.4, 145.6, 148.0, 158.6, 168.7. Anal. for C_13_H_13_N_3_O_5_S: C, 48.29 %; H, 4.05 %; N, 13.00 %; Found: C, 48.32 %; H, 4.00 %; N, 13.20 %.

**N‐(2‐Oxo‐2‐((4‐sulfamoylphenyl)amino)ethyl)thiophene‐2‐carboxamide (32)** Yield: 21 %; m.p.: 265–267 °C; 1H‐NMR (500 MHz, DMSO‐d6): (δ) 4.08 (d, J=5.96 Hz, 2H, CH2), 7.17‐7.19 (m, 1H, ArH), 7.26 (bs, 2H, NH2), 7.77 (bs, 4H, ArH), 7.78‐7.84 (m, 2H, ArH), 8.94 (t, J=5.96 Hz, 1H, NH), 10.49 (s, 1H, NH); Anal. for C_13_H_13_N_3_O_4_S_2_: C, 46.01 %; H, 3.86 %; N, 12.38 %; Found: C, 46.11 %; H, 3.96 %; N, 12.46 %;

### General synthetic procedures for the 2‐(hetero)arylformamido‐N‐[(4‐sulfamoylphenyl]methyl)alkylamides (36‐44)

To a solution of the appropriate aroyl amino acid derivatives (**25‐30** and **33–35**) in N,N‐dimethylformamide (DMF) (2 mL), N,N,N’,N’‐tetramethyl‐O‐(1H‐benzotriazol‐1‐yl)‐uraniumhexafluorophosphate (HBTU) (1 molar equivalent) was added. The mixture was stirred at room temperature for 15 minutes. Then, DIPEA (2.5 molar equivalents) and 4‐aminomethylbenzenesulfonamide hydrochloride (1 molar equivalent) were added. The reaction mixture was left overnight at room temperature and then quenched with H_2_O (10 mL) and extracted with EtOAc (3×10 mL). The organic phase was washed with saturated NaCl solution, dried with Na_2_SO_4_ and concentrated until dryness under reduced pressure. The crude was purified by crystallization from EtOH to give the desired final compounds **36–44** as white powder.

**N‐(2‐Oxo‐2‐((4‐sulfamoylbenzyl)amino)ethyl)benzamide (36)** Yield: 81 %; m.p.: 211–213 °C; ^1^H‐NMR (500 MHz, DMSO‐d_6_): 3.89–3.97 (m, 2H, CH_2_), 4.32–4.40 (m, 2H, CH_2_), 7.31 (bs, 2H, NH_2_), 7.43–7.54 (5H, ArH), 7.75 (d, J=7.25 Hz, 2H, ArH), 7.90(d, J=7.25 Hz, 2H, ArH), 8.55–8.63 (m, 1H, NH), 8.82–8.90 (m, 1H, NH); ^13^C‐NMR (126 MHz, DMSO‐d_6_): (δ) 42.1, 43.2, 126.0, 127.8, 128.6, 131.7, 134.4, 142.9, 144.0, 166.9, 169.7. Anal. for C_16_H_17_N_3_O_4_S: C 55.32 %; H, 4.93 %; N 12.10 %. Found: C 55.23 %; H 4.81 %; N 12.24 %.

**(S)‐N‐(1‐Oxo‐1‐((4‐sulfamoylbenzyl)amino)propan‐2‐yl)benzamide (37)** Yield: 67 %; m.p.: 178–180 °C; ^1^H‐NMR (500 MHz, DMSO‐d_6_): 1.34–1.42 (m, 3H, CH_3_), 4.32–4.42 (m, 1H, CH), 4.48–4.63 (m, 2H, CH_2_), 7.30 (bs, 2H, NH_2_), 7.42–7.54 (m, 5H, ArH), 7.74–7.84 (m, 2H, ArH), 7.88–7.98 (m, 2H, ArH), 8.22–8.65 (m, 1H, NH), 9.08–9.22 (m, 1H, NH); ^13^C‐NMR (126 MHz, DMSO‐d_6_): 18.3, 42.8, 49.7, 126.1, 127.7, 128.0, 128.6, 128.8, 131.8, 134.5, 143.0, 145.0, 166.8, 173.1. Anal. for C_17_H_19_N_3_O_4_S: C 56.50 %; H 5.30 %; N, 11.63 %. Found: C 56.40 %; H 5.43 %; N 11.51 %.

**(S)‐N‐(3‐Methyl‐1‐oxo‐1‐((4‐sulfamoylbenzyl)amino)butan‐2‐yl)benzamide (38**) Yield: 71 %; m.p.: 213–215 °C; ^1^H‐NMR (500 MHz, DMSO‐d_6_): 0.91 (d, J=6.43 Hz, 3H, CH_3_), 0.94 (d, J=6.43 Hz, 3H, CH_3_), 2.13‐2.20 (m, 1H, CH), 4.31 (t, J=8.10 Hz, 16.6 Hz, 1H, CH), 4.37 (d, J=5.97 Hz, 2H, CH_2_), 7.30 (s, 2H, NH_2_), 7.43‐7.56 (m, 5H, ArH), 7.74 (d, J=8.52 Hz, 2H, ArH), 7.89 (d, J=8.52 Hz, 2H, ArH), 8.31 (d, J=8.10, 1H, NH), 8.65 (t, J=5.97, J=11.94 Hz 1H, NH); ^13^C‐NMR (126 MHz, DMSO‐d_6_): 19.3, 19.8, 30.3, 42.2, 59.8, 126.0, 127.9, 128.0, 128.6, 131.7, 134.6, 143.0, 144.0, 167.1, 172.0. Anal. for C_19_H_23_N_3_O_4_S: C 58.59 %; H 5.95 %; N 10.79 %. Found: C 58.47 %; H 6.03 %; N 10.66 %.

**N‐(2‐Oxo‐2‐((4‐sulfamoylbenzyl)amino)ethyl)furan‐2‐carboxamide (39)** Yield: 61 %; m.p.: 200–202 °C; ^1^H‐NMR (500 MHz, DMSO‐d_6_): 3.87 (d, J=6.13 Hz, 2H, CH_2_), 4.34 (d, J=6.89 Hz, 2H, CH_2_), 6.63–6.64 (m, 1H, ArH), 7.14–7.15 (m, 1H, ArH), 7.31 (bs, 2H, NH_2_), 7.43 (d, J=7.97 Hz, 2H, ArH), 7.75 (d, J=7.97 Hz, 2H, ArH), 7.86‐7.88 (m, 1H, ArH), 8.56 (t, J=6.03 Hz, 1H, NH), 8.60 (t, J=5.82 Hz, 1H, NH); ^13^C‐NMR (126 MHz, DMSO‐d_6_): 41.7, 42.1, 111.9, 113.7, 125.6, 127.5, 142.6, 143.6, 145.1, 147.8, 158.2, 169.1. Anal. for C_23_H_23_N_3_O_4_S: C 63.14 %; H 5.30 %; N 9.60 %; Found: C, 63.28 %; H 5.18 %; N 9.72 %.

(**S)‐N‐(1‐Oxo‐1‐((4‐sulfamoylbenzyl)amino)propan‐2‐yl)furan‐2‐carboxamide (40)** Yield: 30 %; m.p.: 215–218 °C; ^1^H‐NMR (500 MHz, DMSO‐d_6_): 1.37 (d, J=7.12 Hz, 3H, CH_3_); 4.35 (d, J=6.07 Hz, 2H, CH_2_), 4.48 (t, J=7.17 Hz, 1H, CH), 6.63–6.64 (m, 1H, ArH), 7.24–7.25 (m, 1H, ArH), 7.29 (s, 2H, NH_2_), 7.43 (d, J=8.38 Hz, 2H, ArH), 7.74 (d, J=8.38 Hz, 2H, ArH), 7.85‐7.86 (m, 1H, ArH), 8.02 (d, J=8.67 Hz, 1H, NH), 8.70 (t, J=5.95 Hz, 1H, NH); ^13^C‐NMR (126 MHz, DMSO‐d_6_): 17.4, 41.7, 49.0, 111.8, 113.8, 125.6, 127.5, 142.6, 143.6, 145.1, 147.7, 158.2, 169.1. Anal. for C_23_H_23_N_3_O_4_S: C, 63.14 %; H, 5.30 %; N, 9.60 %; Found: C, 63.28 %; H, 5.18 %; N, 9.72 %.

**(S)‐N‐(3‐Methyl‐1‐oxo‐1‐((4‐sulfamoylbenzyl)amino)butan‐2‐yl)furan‐2‐carboxamide (41)** Yield: 60 %; m.p.: 226–228 °C; ^1^H‐NMR (500 MHz, DMSO‐d_6_): 0.88 (d, J=2.46 Hz, 3H, CH_3_), 0.89 (d, J=2.46 Hz, 3H, CH_3_), 2.09–2.15 (m, 1H, CH), 4.28 (t, J=8.32 Hz, 1H, CH), 4.36 (d, J=5.94 Hz, 2H, CH_2_), 6.63–6.64 (m, 1H, ArH), 7.24–7.25 (m, 1H, ArH), 7.29 (s, 2H, NH_2_), 7.43 (d, J=8.38 Hz, 2H, ArH), 7.74 (d, J=8.38 Hz, 2H, ArH), 7.85–7.86 (m, 1H, ArH), 8.02 (d, J=8.67 Hz, 1H, NH), 8.70 (t, J=5.95 Hz, 1H, NH); ^13^C‐NMR (126 MHz, DMSO‐d_6_): 18.9, 19.6, 30.4, 42.0, 58.4, 112.0, 114.1, 125.8, 127.7, 142.9, 143.6, 145.4, 147.6, 157.8, 171.2. Anal. for C_17_H_21_N_3_O_5_S: C, 53.18 %; H, 5.58 %; N, 11.07 %; Found: C, 53.31 %; H, 5.45 %; N, 11.18 %.

**N‐(2‐Oxo‐2‐((4‐sulfamoylbenzyl)amino)ethyl)thiophene‐2‐carboxamide (42)** Yield: 40 %; m.p.: 220–222 °C; ^1^H‐NMR (500 MHz, DMSO‐d_6_): 3.85 (d, J=5.94 Hz, 2H, CH_2_), 4.30 (d, J=6.50 Hz, 2H, CH_2_), 7.15 (t, J=4.36 Hz, 1H, ArH), 7.33 (bs, 2H, NH_2_), 7.40 (d, 2H, ArH), 7.68–7.80 (m, 3H, ArH), 7.86 (m, 1H, ArH), 8.67 (m, 1H, NH), 8.97 (m, 1H, NH); ^13^C‐NMR (126 MHz, DMSO‐d_6_): 42.0, 42.8, 125.9, 127.7, 128.2, 128.9, 131.1, 139.9, 142.8, 143.9, 161.8, 169.4. Anal. for C_14_H_15_N_3_O_4_S_2_: C, 47.58 %; H, 4.28 %; N, 11.89 %; Found: C, 47.43 %; H, 4.40 %; N, 12.00 %.

**(S)‐N‐(1‐Oxo‐1‐((4‐sulfamoylbenzyl)amino)propan‐2‐yl)thiophene‐2‐carboxamide (43)** Yield: 57 %; m.p.: 221–223 °C; ^1^H‐NMR (500 MHz, DMSO‐d_6_): 1.37 (d, J=7.12 Hz, 3H, CH_3_); 4.35 (d, J=6.07 Hz, 2H, CH_2_), 4.48 (t, J=7.17 Hz, 1H, CH), 7.16 (t, 1H, ArH); 7.30 (bs, 2H, NH_2_), 7.43 (d, 2H, ArH); 7.73 (m, 3H, ArH), 7.91 (d, 1H, ArH), 8.54 (m, 2H, NH); ^13^C‐NMR (126 MHz, DMSO‐d_6_): 18.2, 42.2, 49.7, 126.2, 127.9, 128.4, 129.3, 131.6, 139.7, 142.7, 144.1, 161.6, 173.0. Anal. for C_15_H_17_N_3_O_4_S_2_: C, 49.03 %; H, 4.66 %; N, 11.44 %; Found: C, 49.17 %; H, 4.51 %; N, 11.56 %.

**(S)‐N‐(3‐Methyl‐1‐oxo‐1‐((4‐sulfamoylbenzyl)amino)butan‐2‐yl)thiophene‐2‐carboxamide (44)** Yield: 64 %; m.p.: 144–146 °C; ^1^H‐NMR (500 MHz, DMSO‐d_6_): 0.89 (d, J=6.63 Hz, 3H, CH_3_), 0.92 (d, J=6.62 Hz, 3H, CH_3_), 2.09–2.18 (m, 1H, CH), 4.26 (t, J=8.54 Hz, 1H, CH), 4.36 (d, J=5.86 Hz, 2H, CH_2_), 7.16 (m, 1H, ArH), 7.30 (s, 2H, NH_2_), 7.43 (d, J=8.25 Hz, 2H, ArH), 7.72–7.79 (m, 3H, ArH), 7.97 (d, 1H, ArH), 8.38 (d, J=8.80 Hz, 1H, ArH), 8.69 (t, J=5.94 Hz, 1H, NH);^13^C‐NMR (126 MHz, DMSO‐d_6_): 19.3, 19.8, 30.2, 42.1, 59.6, 126.0, 127.9, 128.3, 129.1, 131.4, 139.9, 142.9, 143.9, 161.6, 171.6. Anal. for C_17_H_21_N_3_O_4_S_2_: C, 51.63 %; H, 5.35 %; N, 10.62 %; Found: C, 51.48 %; H, 5.47 %; N, 10.48 %.

### Preparation of the bacterial CAs

The three bacterial enzymes were prepared accordingly to the procedure reported by our groups.^[33]^ Briefly, the GeneArt Company (Invitrogen), specialized in gene synthesis, designed the genes encoding for the bacterial α, β, and γ‐ CAs. The BL21 DE3 competent cells (Agilent) were transformed with the expression vector pET15‐b containing the gene encoding for one of the three CA‐classes. Subsequently, bacterial cells were induced with 1 mM IPTG and, after 30 min, treated with 0.1 M ZnCl_2_. After 4 h, cells were harvested and disrupted by sonication at 4 °C. After centrifugation at 12,000×g for 45 min, the supernatant was incubated with His Select HF nickel affinity gel resin (Sigma) equilibrated in lysis buffer for 30 min. The protein was eluted with the wash buffer containing 200 mM imidazole. Collected fractions were dialyzed against 50 mM Tris/HCl, pH 8. At this stage of purification, the protein was at least 95 % pure.

### Carbonic anhydrase inhibition assay

An Applied Photophysics stopped‐flow instrument has been used for assaying the CA catalysed CO_2_ hydration activity. ^[34]^ Phenol red (at a concentration of 0.2 mM) has been used as indicator, working at the absorbance maximum of 557 nm, with 10–20 mM Hepes (pH 7.5) or Tris (pH 8.3) as buffers, and 20 mM Na_2_SO_4_ or 20 mM NaClO_4_ (for maintaining constant the ionic strength), following the initial rates of the CA‐catalyzed CO_2_ hydration reaction for a period of 10–100 s. The CO_2_ concentrations ranged from 1.7 to 17 mM for the determination of the kinetic parameters and inhibition constants. For each inhibitor at least six traces of the initial 5–10 % of the reaction have been used for determining the initial velocity. The uncatalyzed rates were determined in the same manner and subtracted from the total observed rates. Stock solutions of inhibitor (10 mM) were prepared in distilled‐deionized water and dilutions up to 0.01 nM were done thereafter with distilled‐deionized water. Inhibitor and enzyme solutions were preincubated together for 15 min at room temperature prior to assay, in order to allow for the formation of the E−I complex. The inhibition constants were obtained by non‐linear least‐squares methods using PRISM 3 and represent the mean from at least three different determinations. CA isoforms were recombinant ones obtained as reported earlier by this group[^10^, ^35^, ^36^]

## Author Contributions

All authors equally contributed to the work. The manuscript was written through contributions of all authors. All authors have given approval to the final version of the manuscript.

## Conflict of interest

The authors declare no conflict of interest.

## Supporting information

As a service to our authors and readers, this journal provides supporting information supplied by the authors. Such materials are peer reviewed and may be re‐organized for online delivery, but are not copy‐edited or typeset. Technical support issues arising from supporting information (other than missing files) should be addressed to the authors.

Supporting Information
