# Supplementary material for: 4‐Sulfamoylphenylalkylamides as Inhibitors of Carbonic Anhydrases Expressed in Vibrio cholerae
Source: ChemMedChem. 2021 Oct 18;16(24):3787–94. doi: 10.1002/cmdc.202100510 (PMC9298201; doi:10.1002/cmdc.202100510)
Supplement: Supplementary file 1 — Supporting Information [file CMDC-16-3787-s001.pdf]

ChemMedChem

Supporting Information

4-Sulfamoylphenylalkylamides as Inhibitors of Carbonic Anhydrases Expressed in *Vibrio cholerae*

Francesca Mancuso, Laura De Luca, Federica Bucolo, Milan Vrabel, Andrea Angeli, Clemente Capasso, Claudiu T. Supuran, and Rosaria Gitto*

Supporting Information

Content:

1. Selected representative ^{1}H-NMR and ^{13}C-NMR spectra
2. Selected HPLC Chromatogram and mass spectra
3. Sequence alignment of targeted CA enzymes

1 Selected representative ^{1}H-NMR and ^{13}C-NMR spectra

Figure S1. ^{1}H-NMR (400 MHz, DMSO-d_6) of (9H-fluoren-9-yl)methyl (2-oxo-2-((4-sulfamoylphenyl)amino)ethyl)carbamate, (**7**)

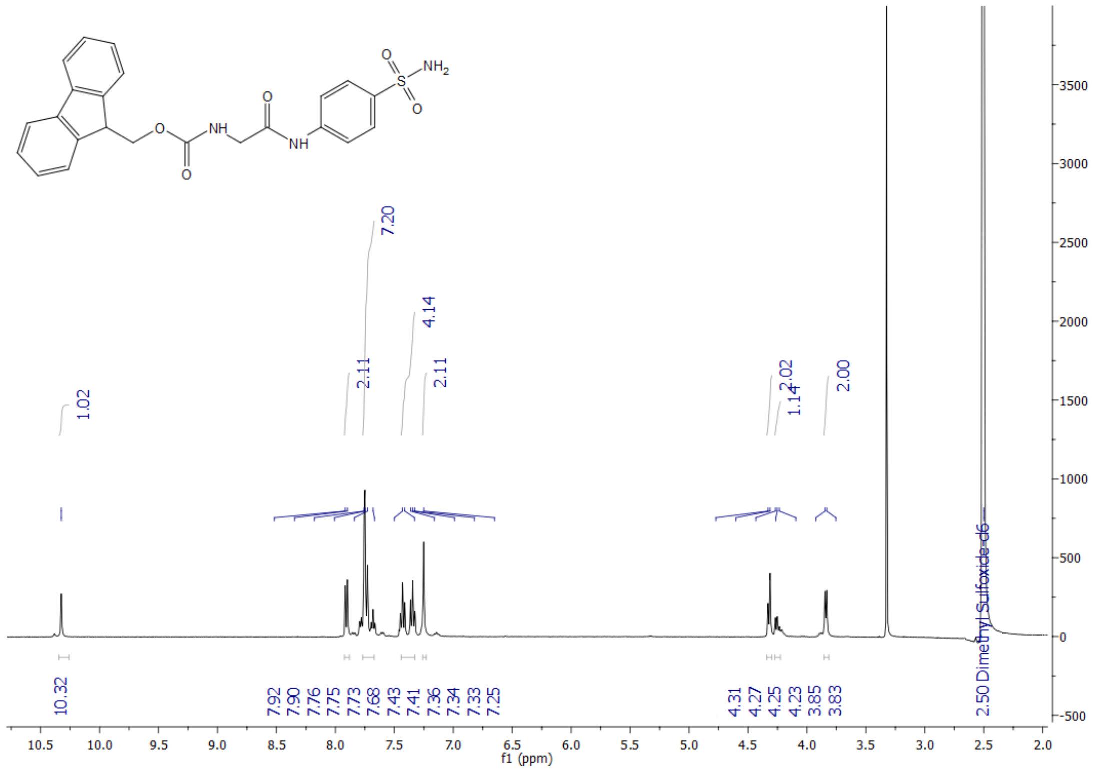

Figure S2. ^{1}H-NMR (400 MHz, DMSO-d_6) of (S)-(9H-fluoren-9-yl)methyl (1-oxo-1-((4-sulfamoylphenyl)amino)propan-2-yl)carbamate, (**8**)

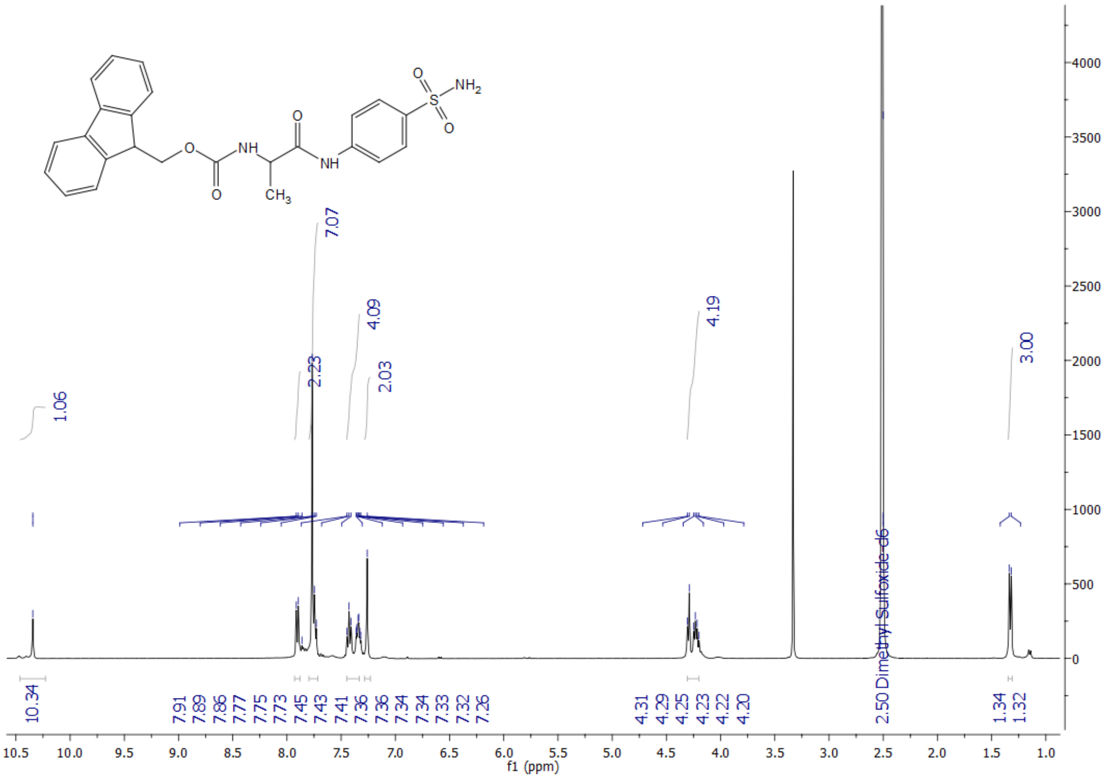

Figure S3. ^{1}H-NMR (400 MHz, DMSO-d_6) of (*S*)-(9*H*-fluoren-9-yl)methyl (3-methyl-1-oxo-1-((4-sulfamoylphenyl)amino)butan-2-yl)carbamate, (**9**)

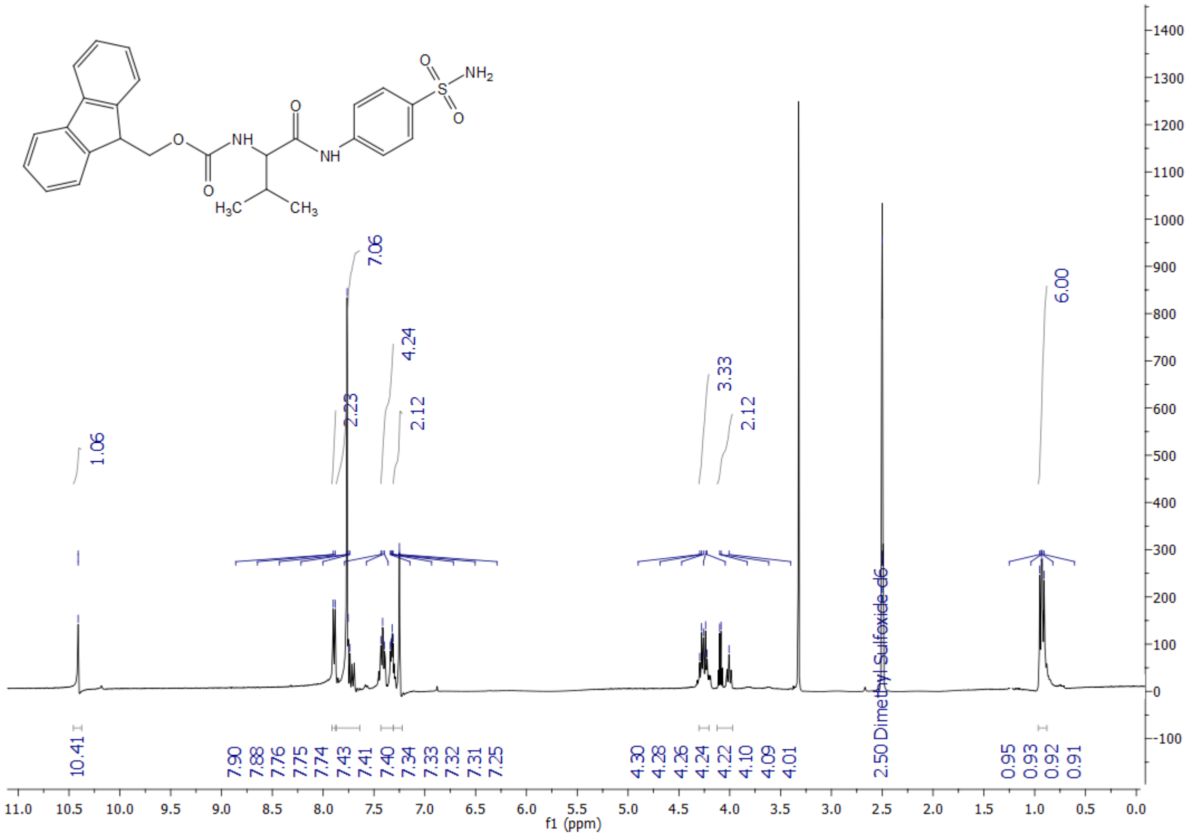

Figure S4. ^{1}H-NMR (400 MHz, Methanol-d_4) of (*S*)-2-amino-N-(4-sulfamoylphenyl)propanamide, (**11**)

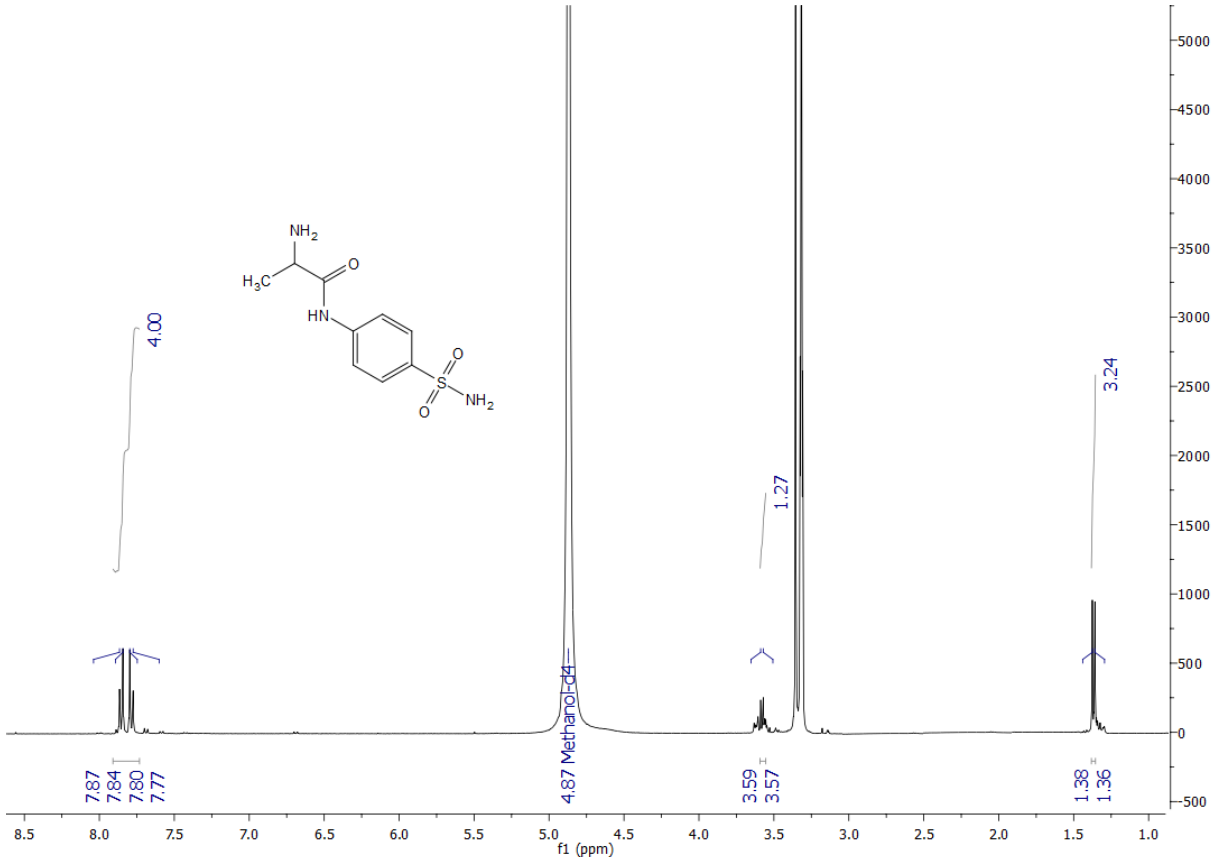

Figure S5. ^{13}C-NMR (400 MHz, DMSO-d_6) of (*S*)-2-amino-N-(4-sulfamoylphenyl)propanamide, (**11**)

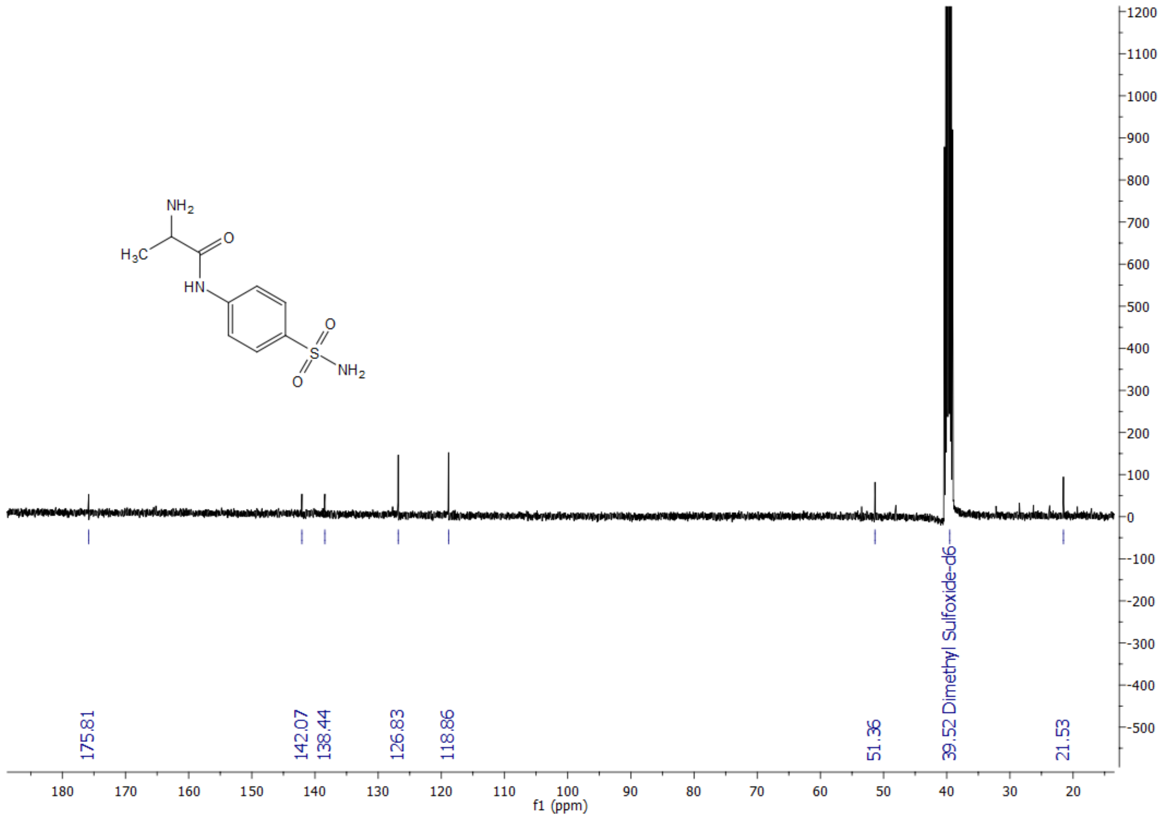

Figure S6. ¹H-NMR (400 MHz, DMSO-*d*₆) of (*S*)-2-amino-3-methyl-N-(4-sulfamoylphenyl)butanamide, (**12**)

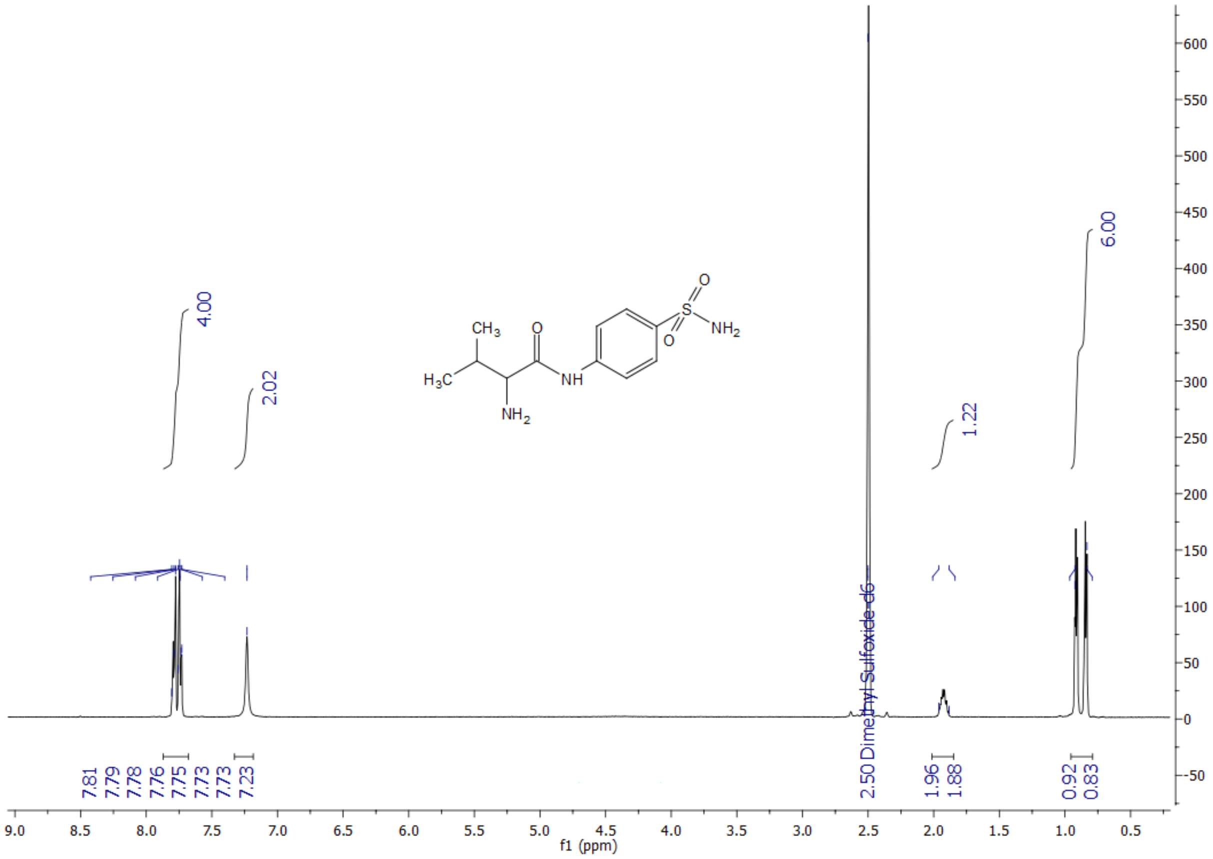

Figure S7. ¹H-NMR (500 MHz, DMSO-*d*₆) of N-2-oxo-2-[(4-sulfamoylphenyl)amino)ethyl]benzamide, (**13**)

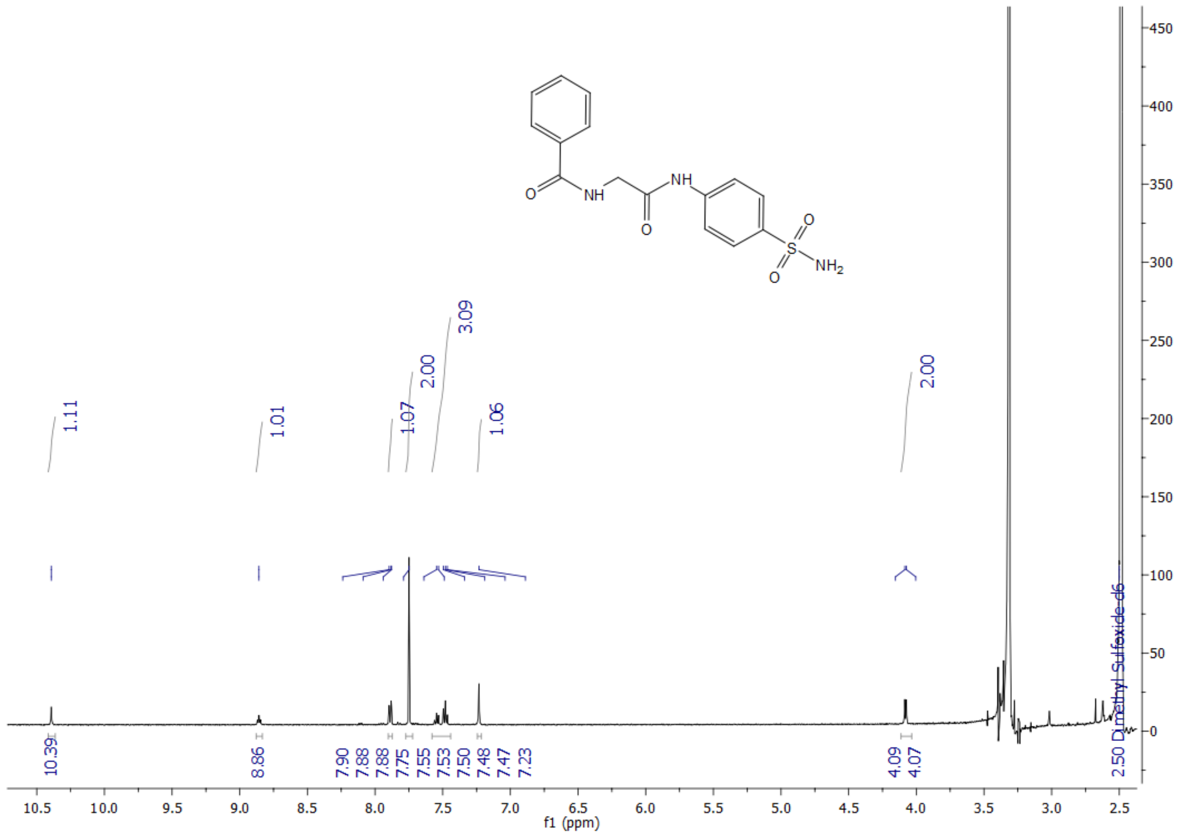

Figure S8. ^{13}C-NMR (126 MHz, DMSO-*d*$_6$) of N-2-oxo-2-[(4-sulfamoylphenyl)amino)ethyl]benzamide, (**13**)

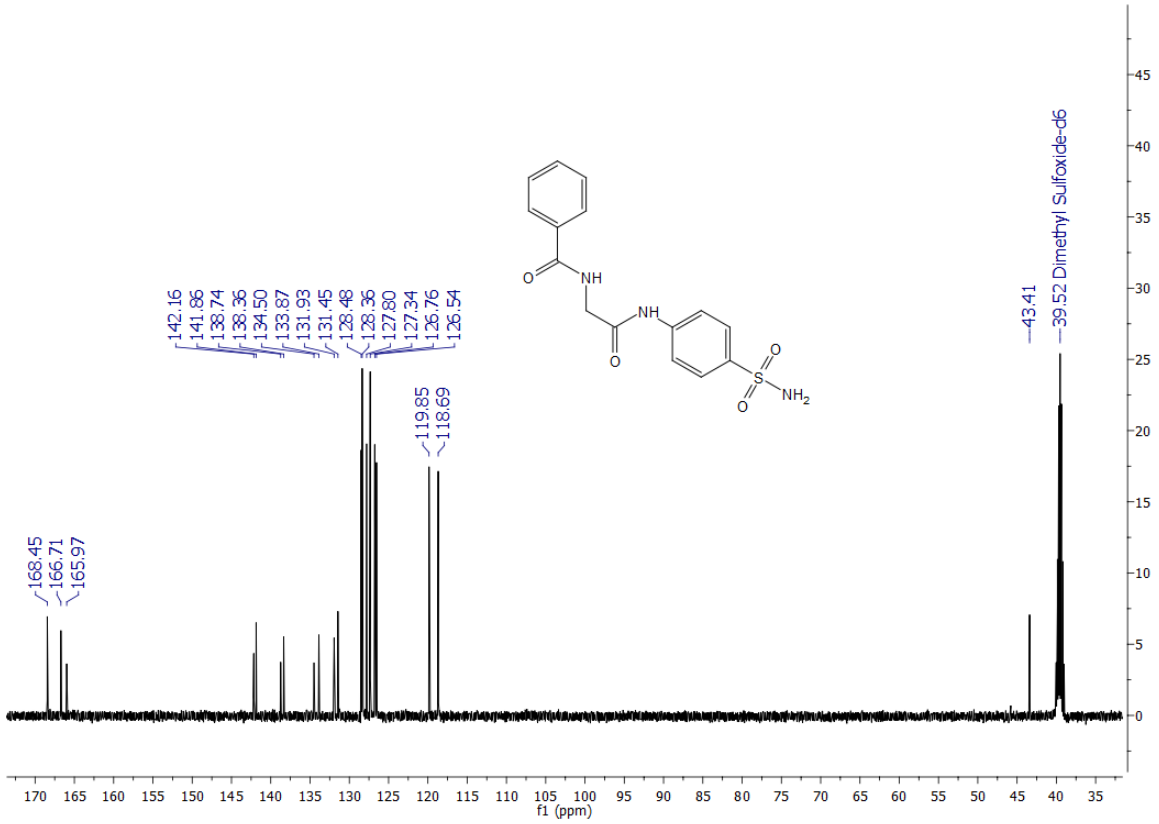

Figure S9. ^{1}H-NMR (500 MHz, DMSO-*d*$_6$) of (*S*)-N-(1-oxo-1-((4-sulfamoylphenyl)amino)propan-2-yl)benzamide, (**14**)

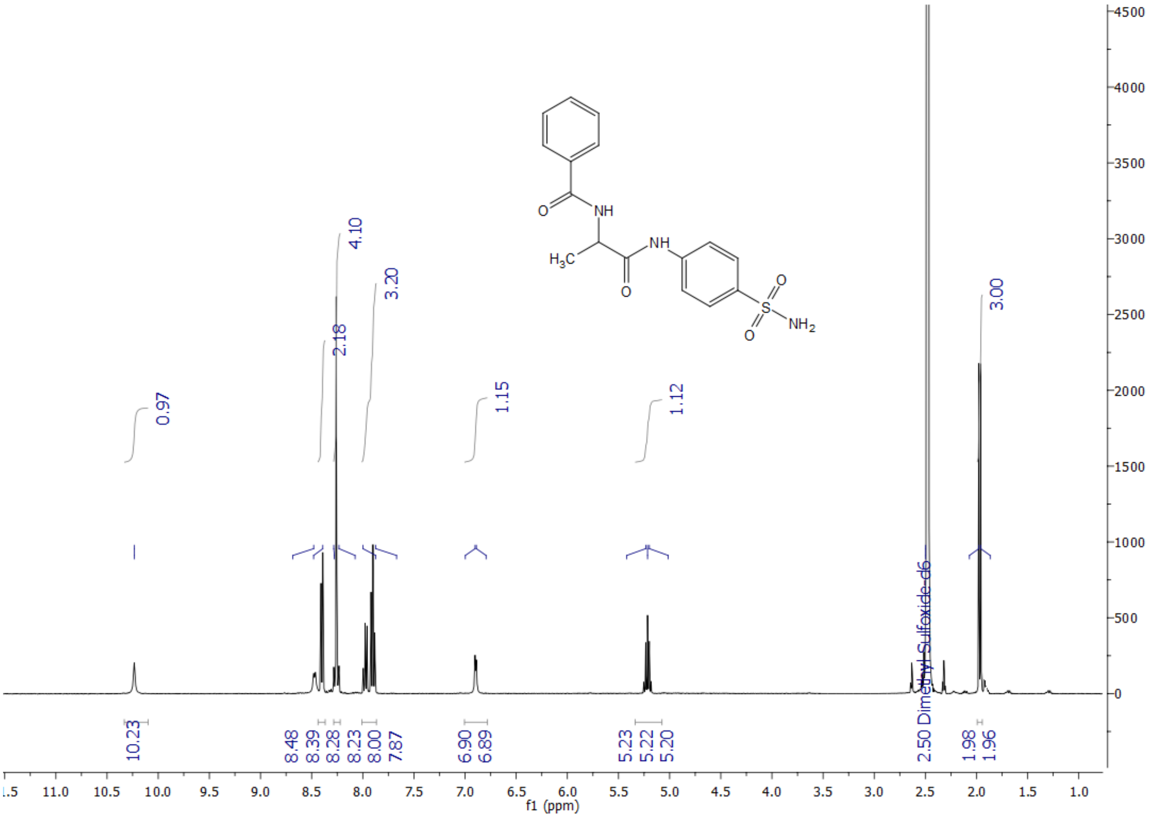

Figure S10. ^{13}C-NMR (126 MHz, DMSO-d_6) Of (*S*)-N-(1-oxo-1-((4-sulfamoylphenyl)amino)propan-2-yl)benzamide, (**14**)

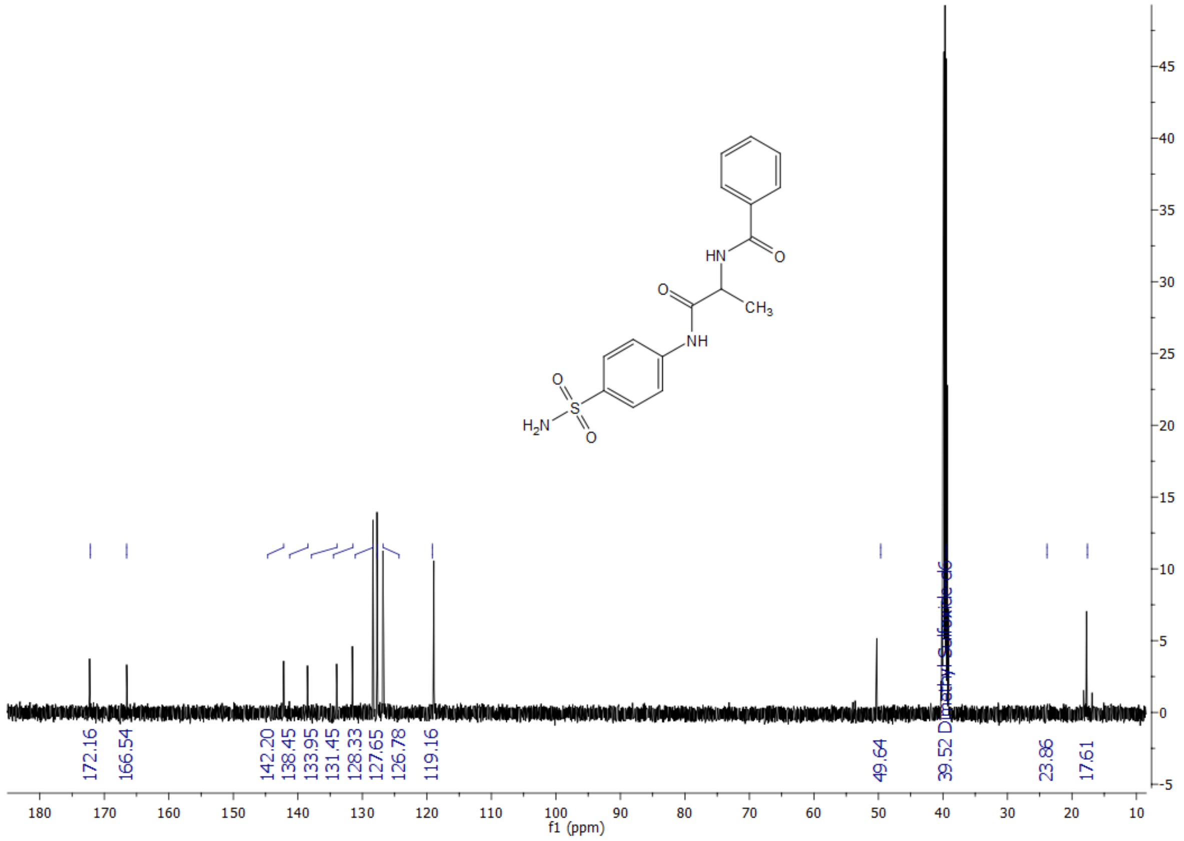

Figure S11. ^{1}H-NMR (500 MHz, DMSO-d_6) of (*S*)-N-(3-methyl-1-oxo-1-((4-sulfamoylphenyl)amino)butan-2-yl)benzamide, (**15**)

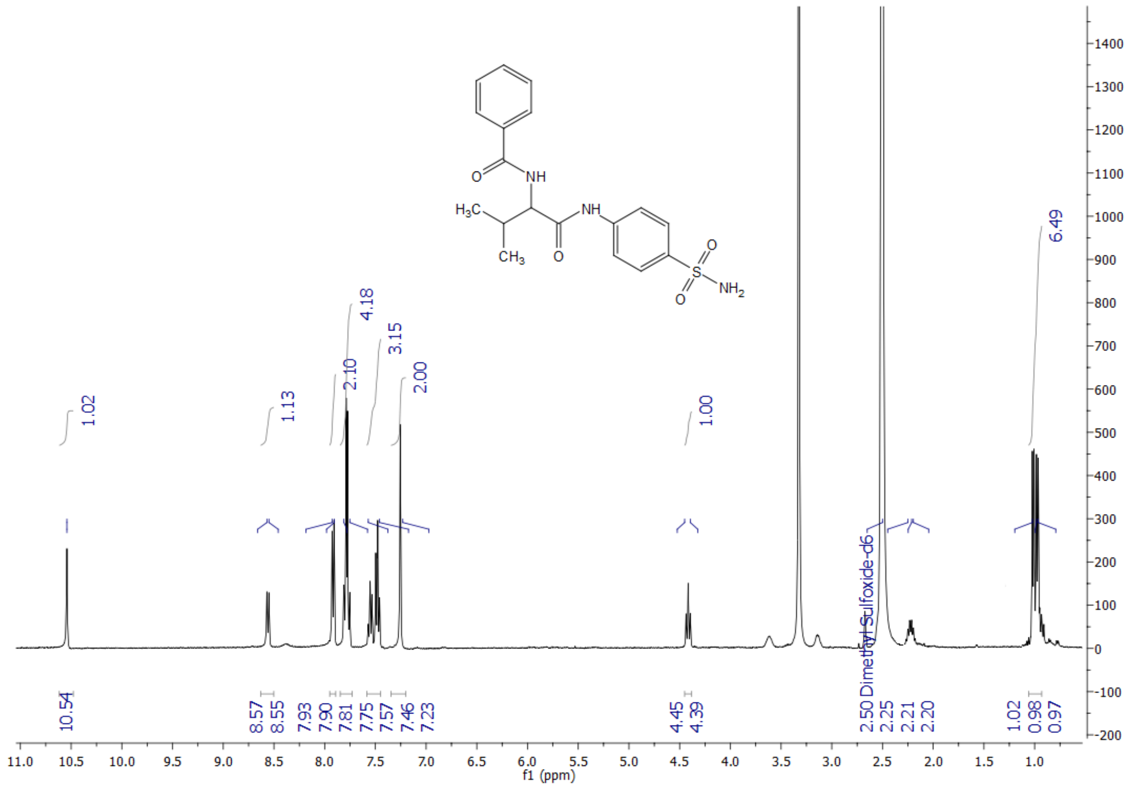

Figure S12. ^{1}H-NMR (500 MHz, DMSO-d_6) of 2-chloro-N-(2-oxo-2-((4-sulfamoylphenyl)amino)ethyl)benzamide, (**20**)

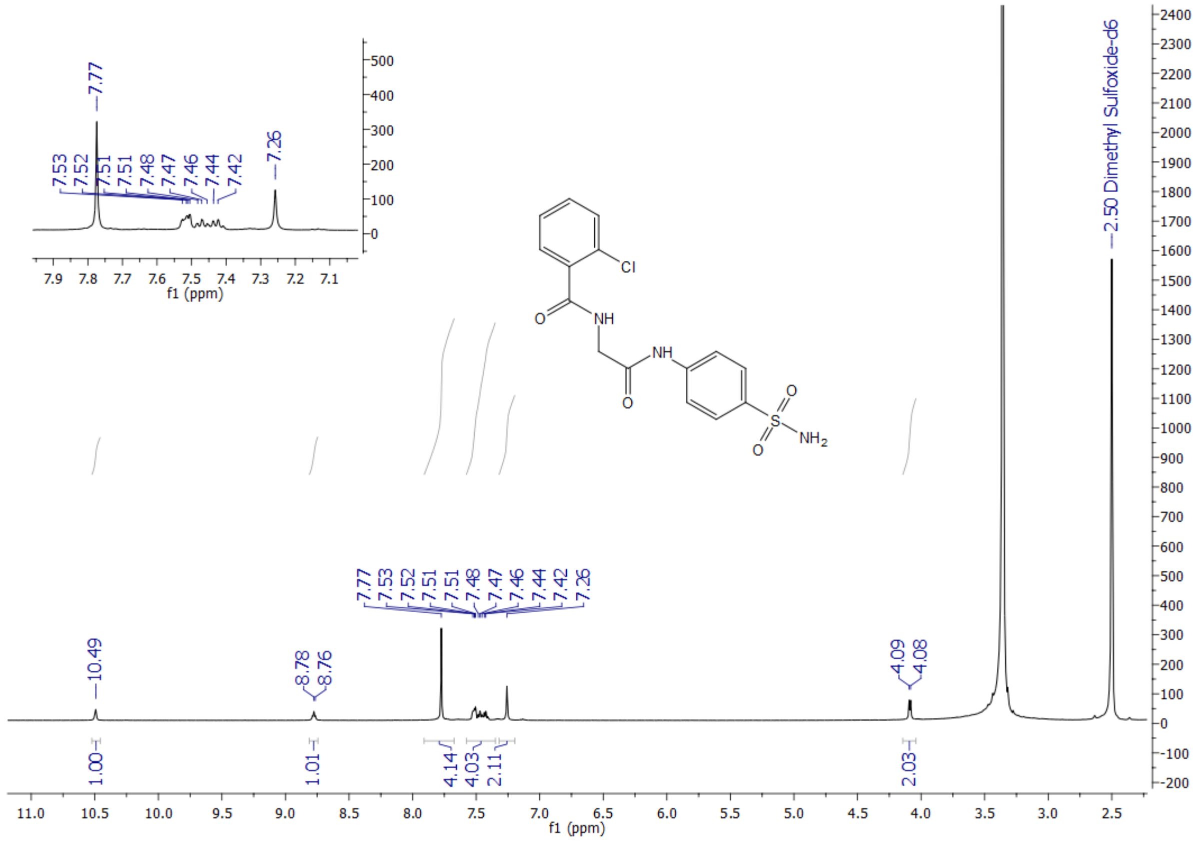

Figure S13. ^{1}H-NMR (500 MHz, DMSO-d_6) of 3-chloro-N-(2-oxo-2-((4-sulfamoylphenyl)amino)ethyl)benzamide, (**21**)

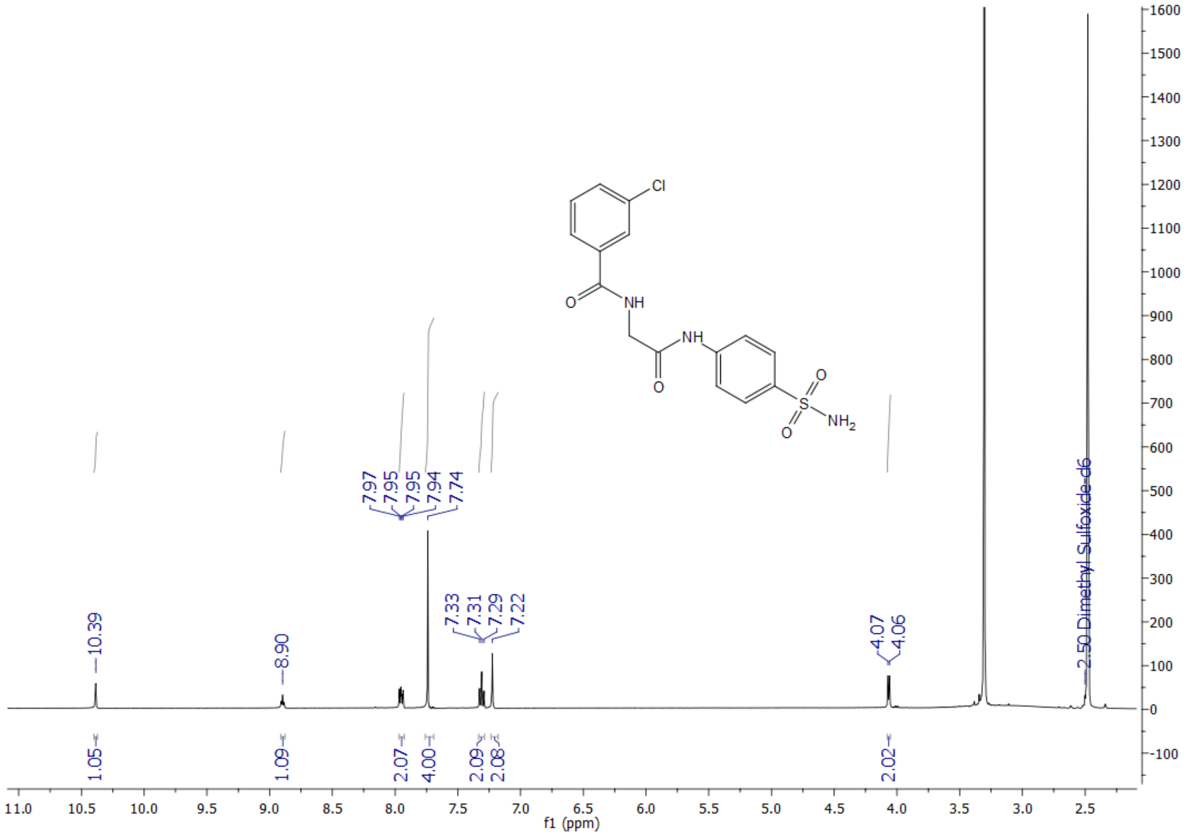

Figure S14. ^{1}H-NMR (500 MHz, DMSO-d_6) of N-(2-oxo-2-((4-sulfamoylphenyl)amino)ethyl)furan-2-carboxamide, (**31**)

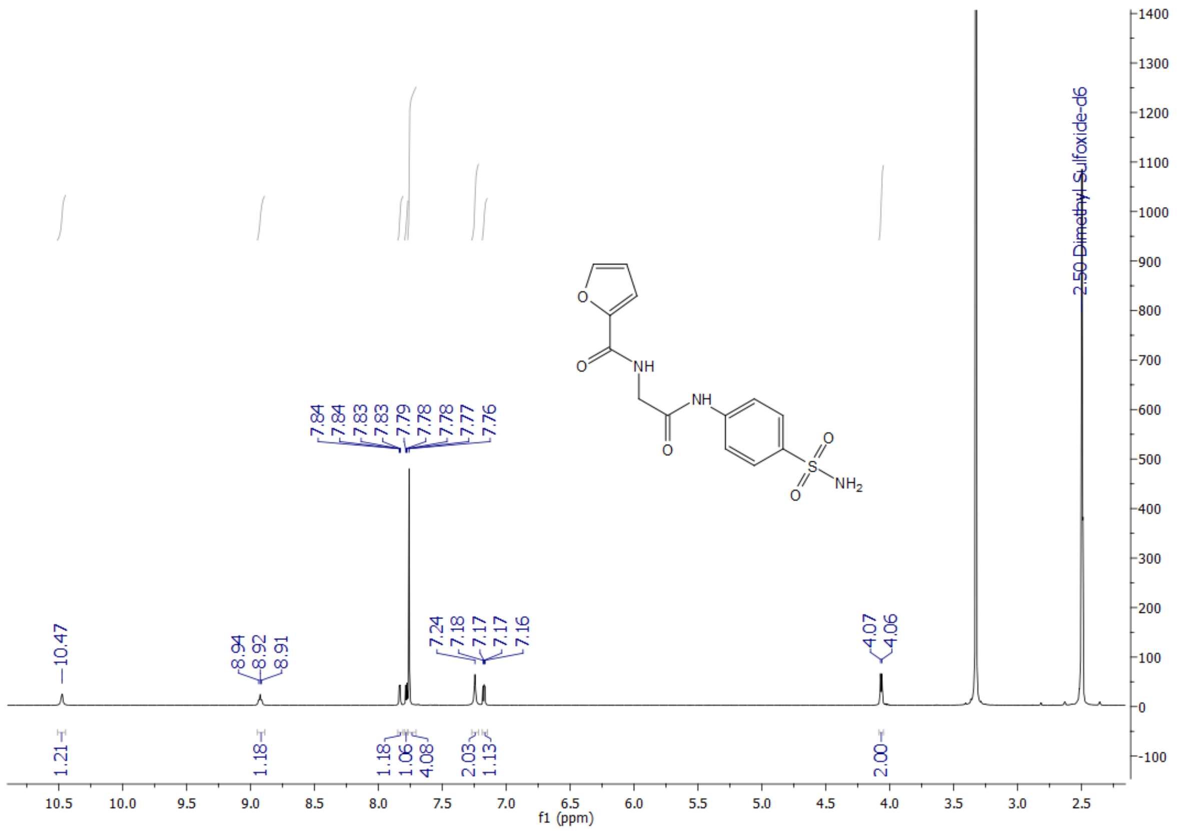

Figure S15. ^{13}C-NMR (126 MHz, DMSO-d_6) of N-(2-oxo-2-((4-sulfamoylphenyl)amino)ethyl)furan-2-carboxamide, (**31**)

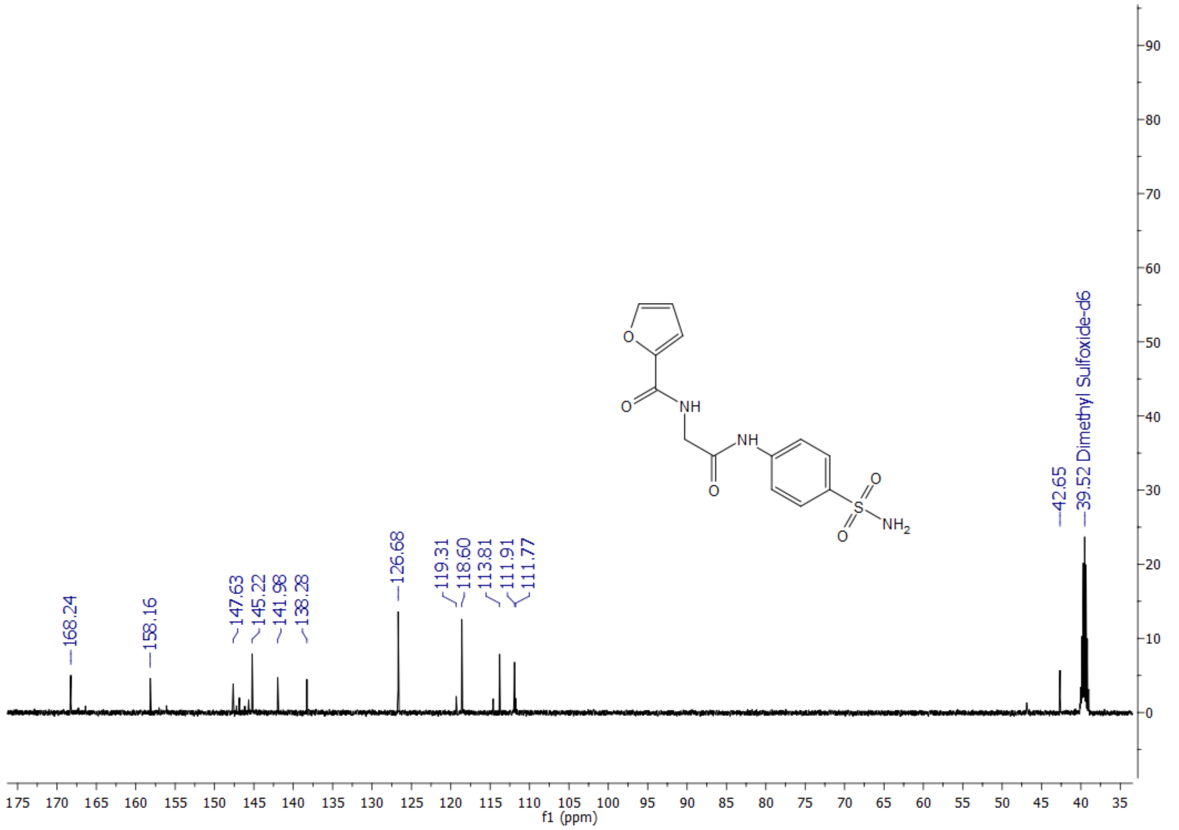

Figure S16. ^{1}H-NMR (500 MHz, DMSO-d_6) of N-(2-oxo-2-((4-sulfamoylphenyl)amino)ethyl)thiophene-2-carboxamide (**32**)

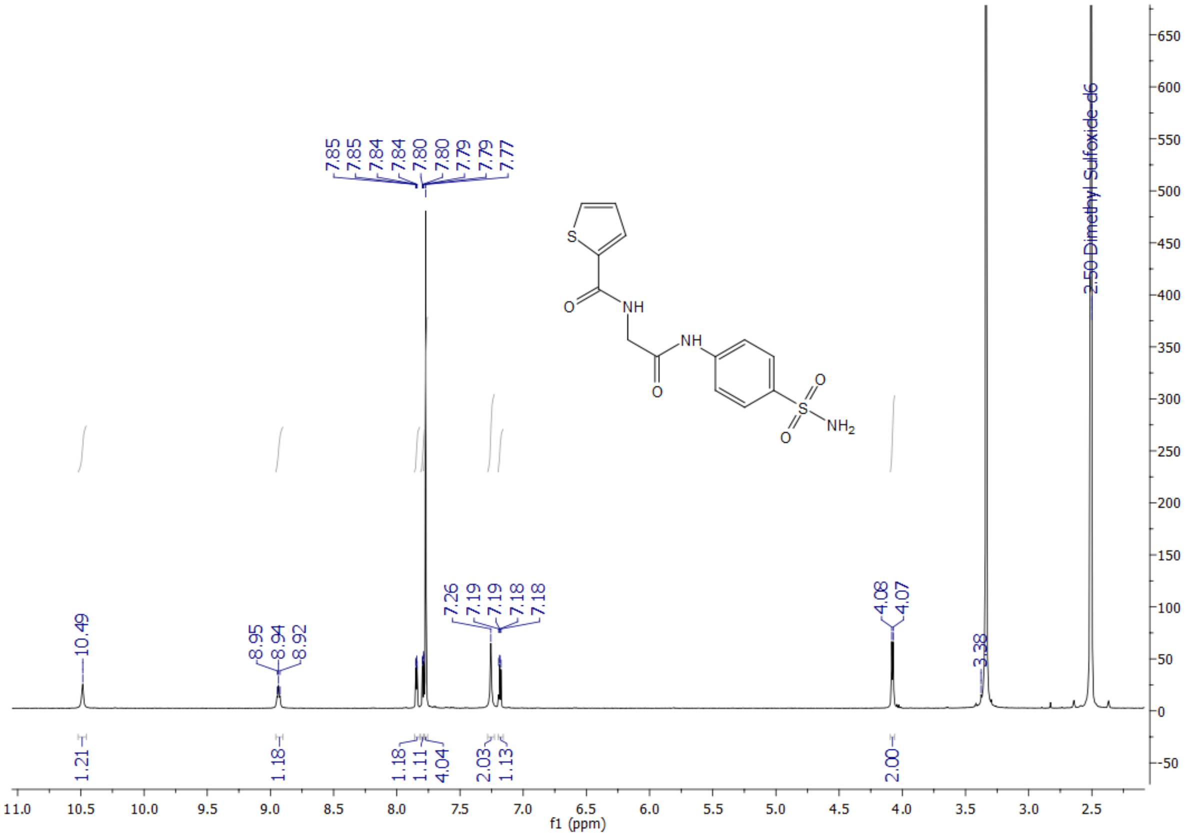

Figure S17. ^{13}C-NMR (126 MHz, DMSO-d_6) of N-(2-oxo-2-((4-sulfamoylphenyl)amino)ethyl)thiophene-2-carboxamide (**32**)

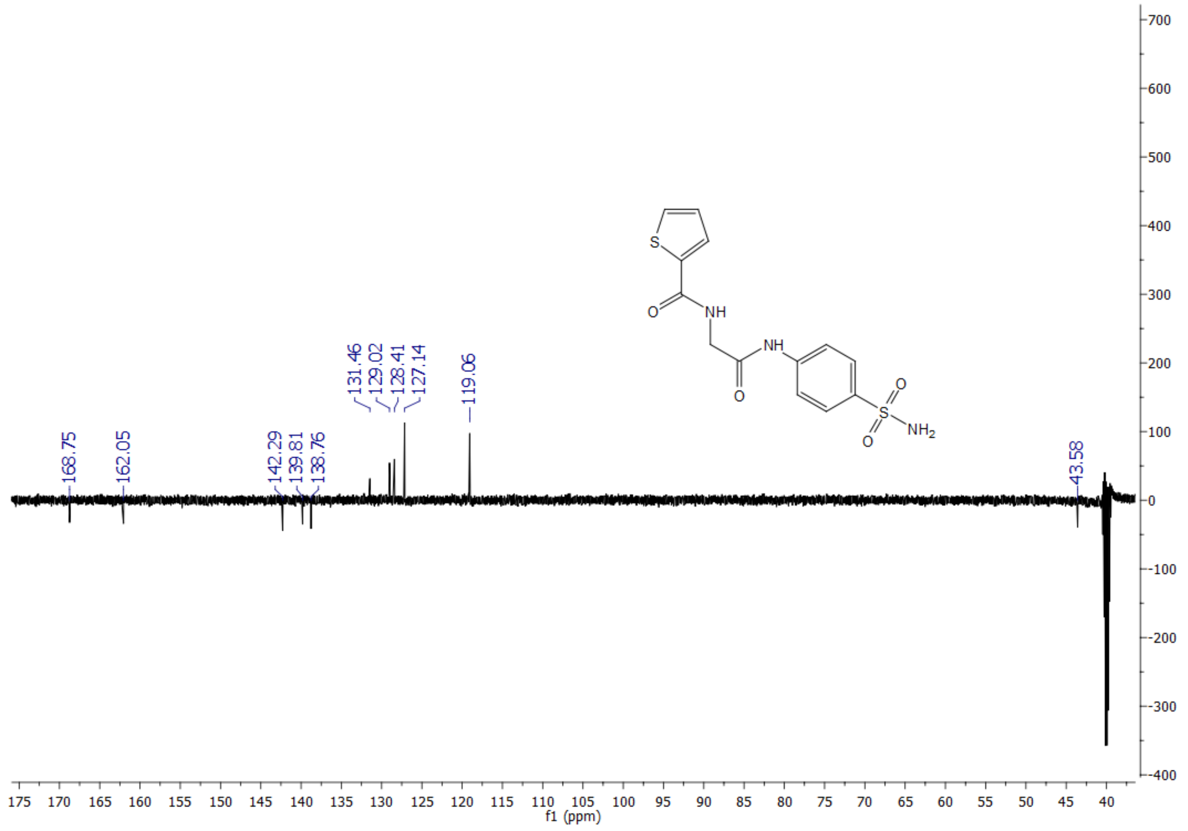

Figure S18. ^{1}H-NMR (500 MHz, DMSO-d_6) of N-(2-oxo-2-((4-sulfamoylbenzyl)amino)ethyl)benzamide, (**36**)

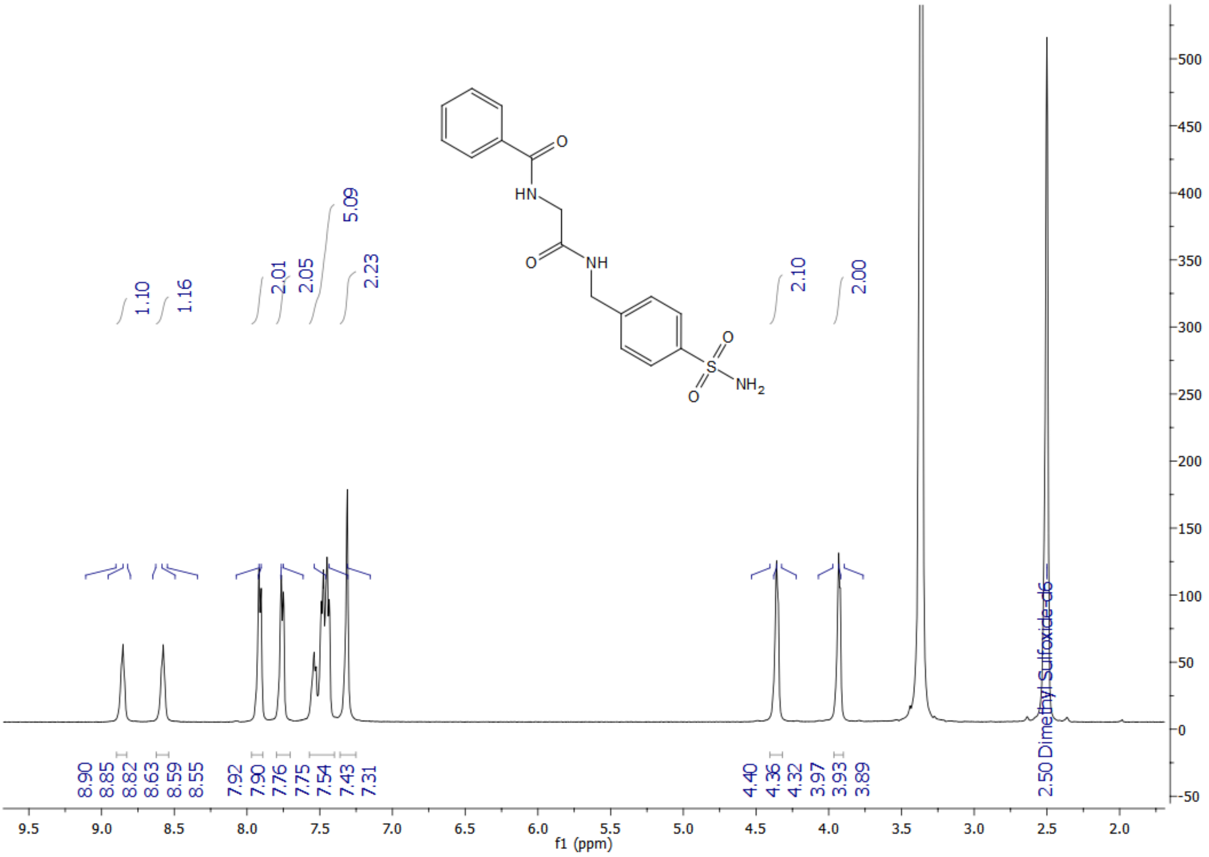

Figure S19. ^{13}C-NMR (126 MHz, DMSO-d_6) of N-(2-oxo-2-((4-sulfamoylbenzyl)amino)ethyl)benzamide, (**36**)

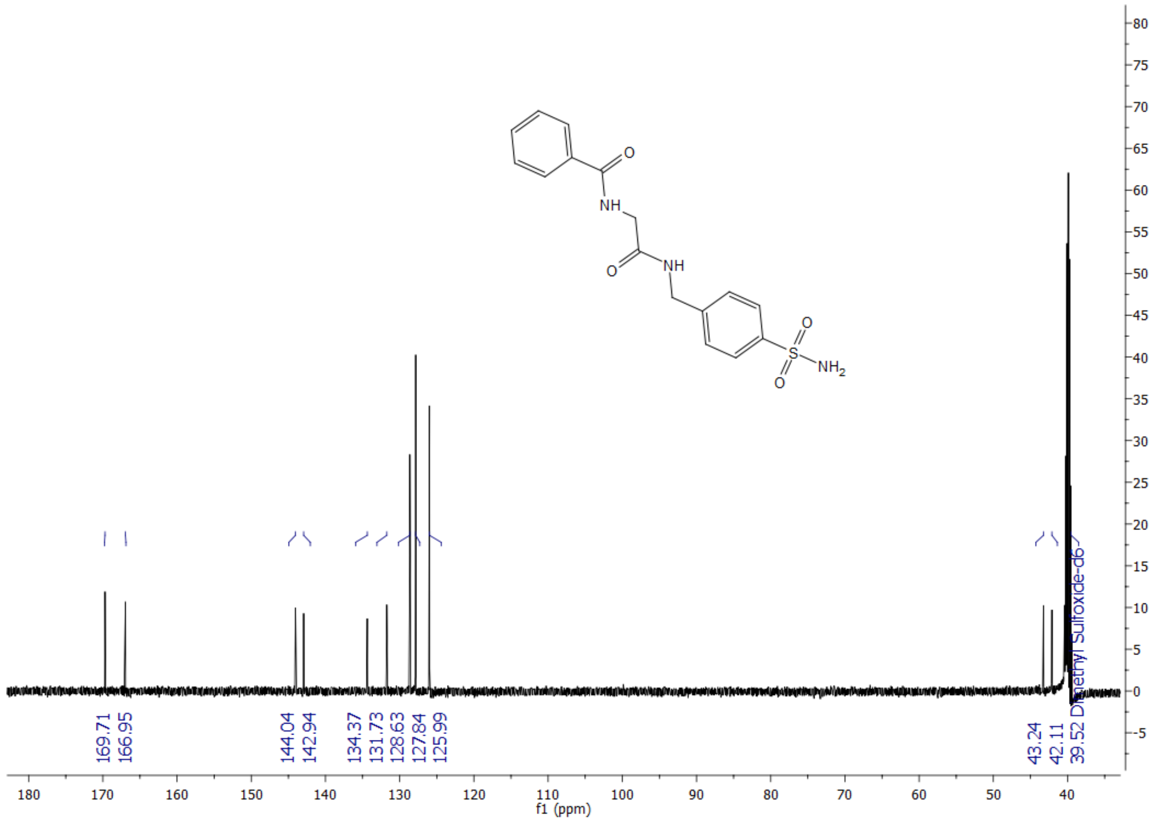

Figure S20. ^{1}H-NMR (500 MHz, DMSO-*d*$_6$) of (*S*)-N-(1-oxo-1-((4-sulfamoylbenzyl)amino)propan-2-yl)benzamide, (**37**)

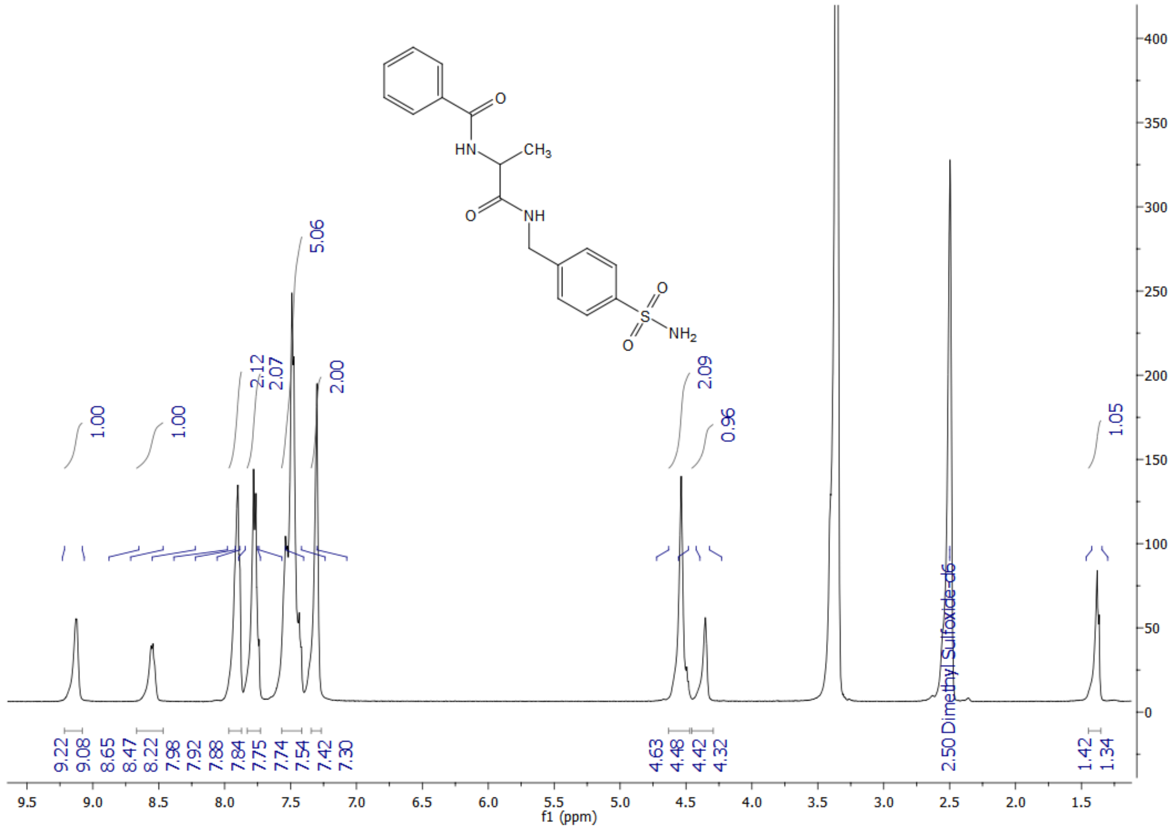

Figure S21. ^{13}C-NMR (126 MHz, DMSO-*d*$_6$) of (*S*)-N-(1-oxo-1-((4-sulfamoylbenzyl)amino)propan-2-yl)benzamide, (**37**)

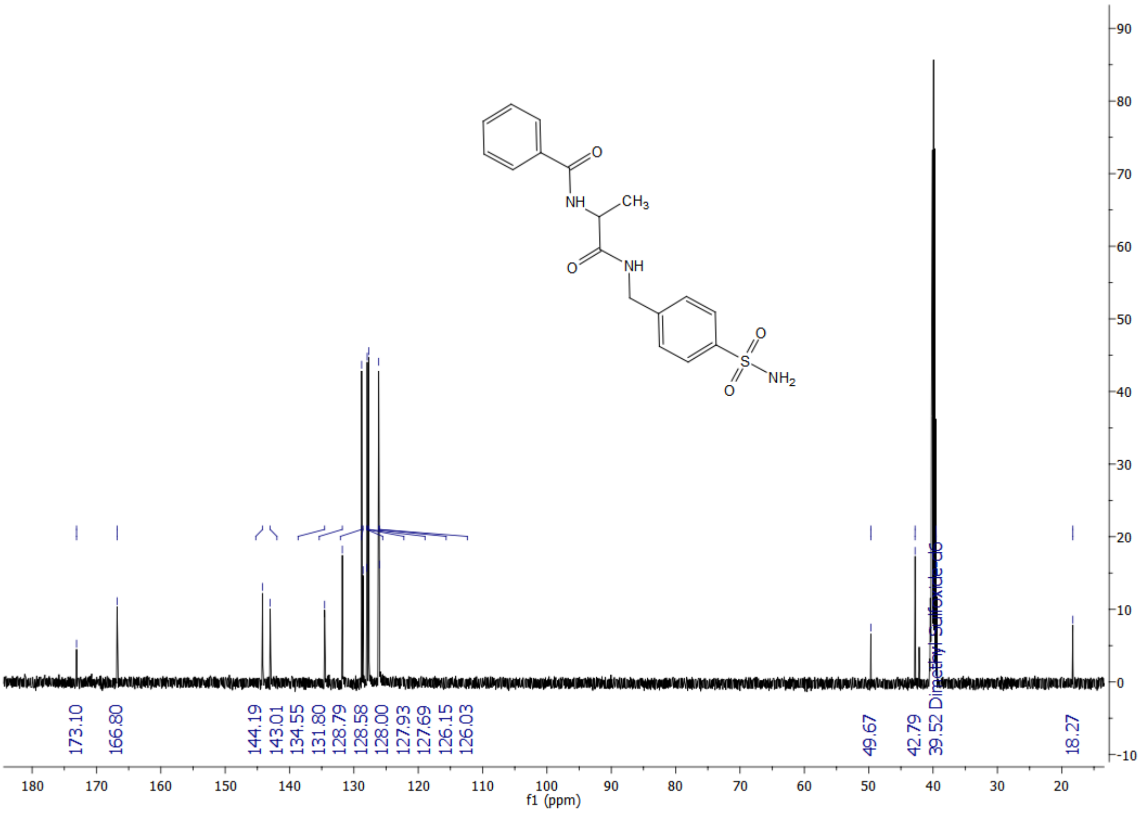

Figure S22. ^{1}H-NMR (500 MHz, DMSO-d_6) of (*S*)-N-(3-methyl-1-oxo-1-((4-sulfamoylbenzyl)amino)butan-2-yl)benzamide, (**38**)

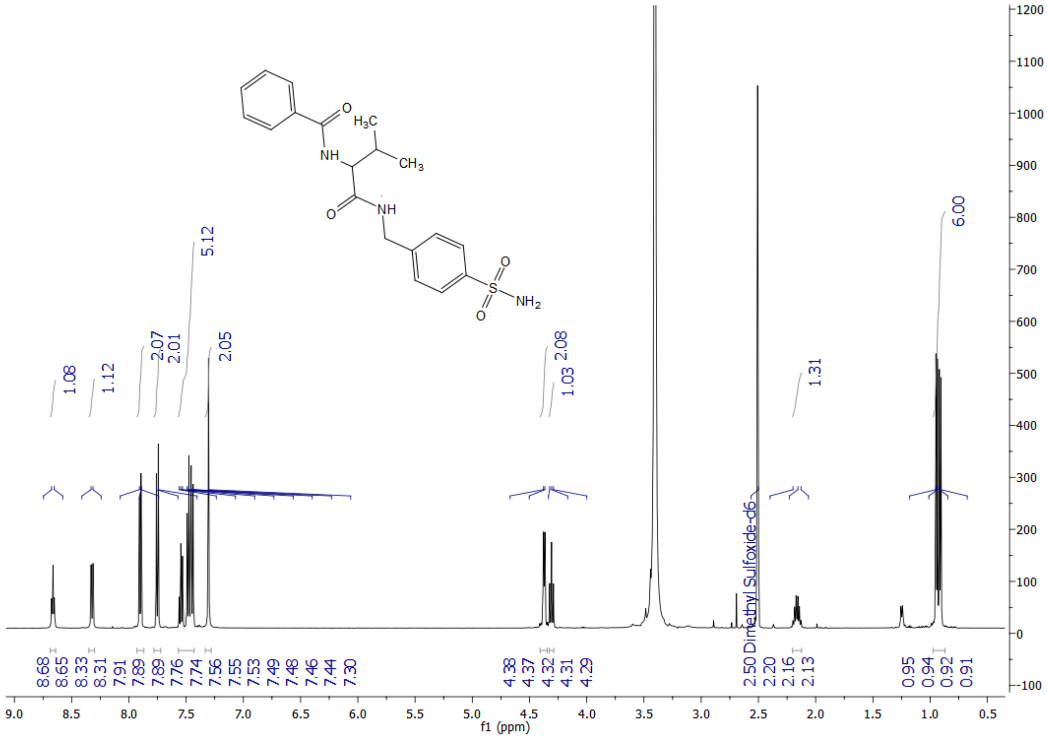

Figure S23. ^{13}C-NMR (126 MHz, DMSO-d_6) of (*S*)-N-(3-methyl-1-oxo-1-((4-sulfamoylbenzyl)amino)butan-2-yl)benzamide, (**38**)

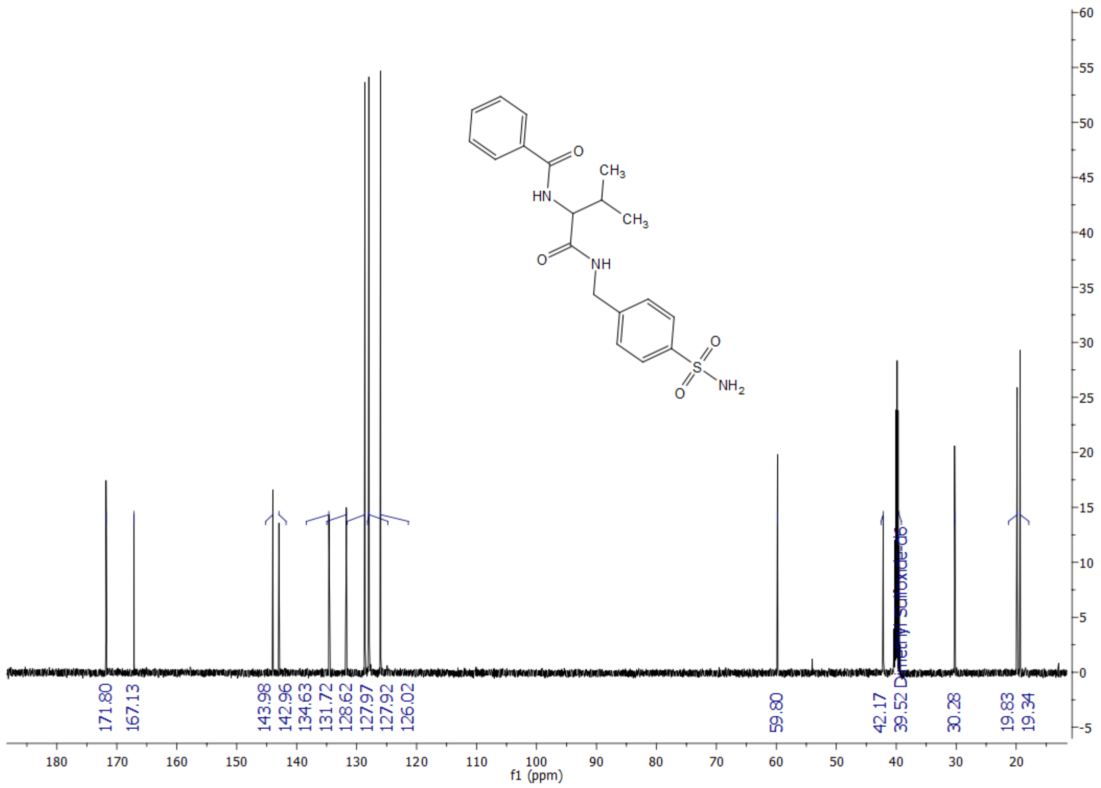

Figure S24. ^{1}H-NMR (500 MHz, DMSO-d_6) of N-(2-oxo-2-((4-sulfamoylbenzyl)amino)ethyl)furan-2-carboxamide, (**39**)

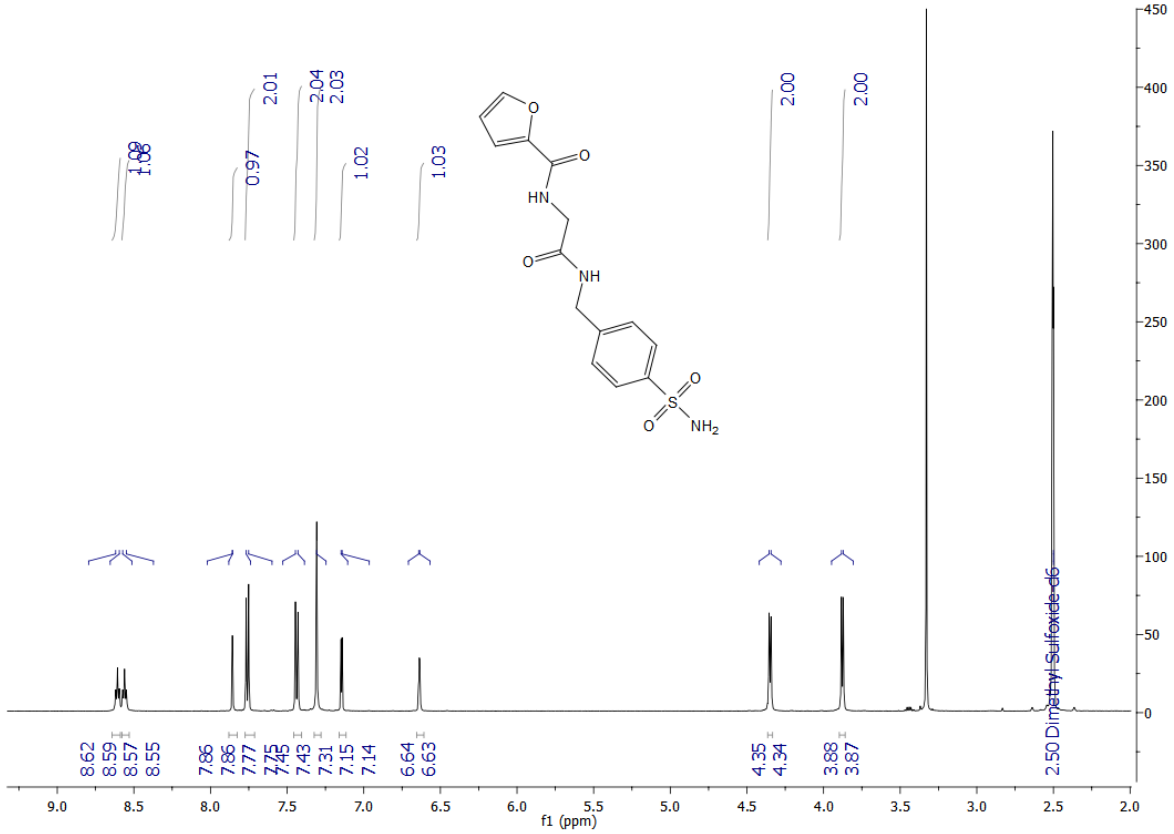

Figure S25. ^{13}C-NMR (126 MHz, DMSO-d_6) of N-(2-oxo-2-((4-sulfamoylbenzyl)amino)ethyl)furan-2-carboxamide, (**39**)

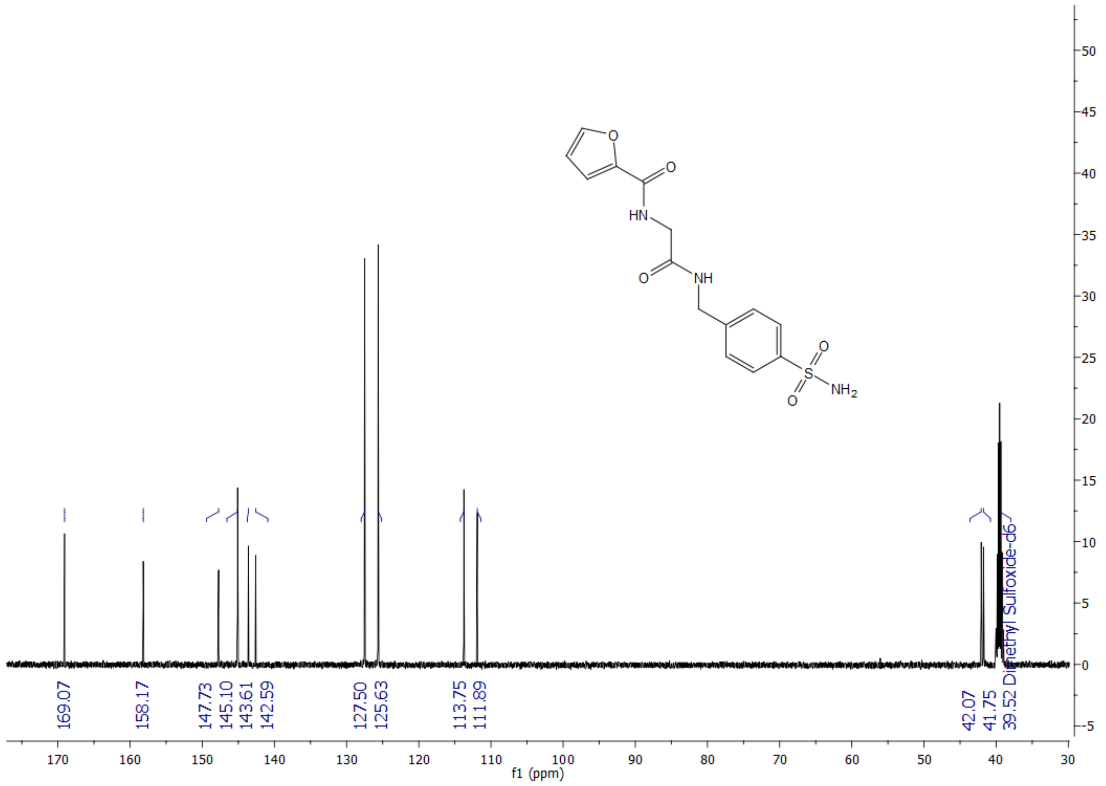

Figure S26. ^{1}H-NMR (500 MHz, DMSO-d_6) of (*S*)-N-(1-oxo-1-((4-sulfamoylbenzyl)amino)propan-2-yl)furan-2-carboxamide, (**40**)

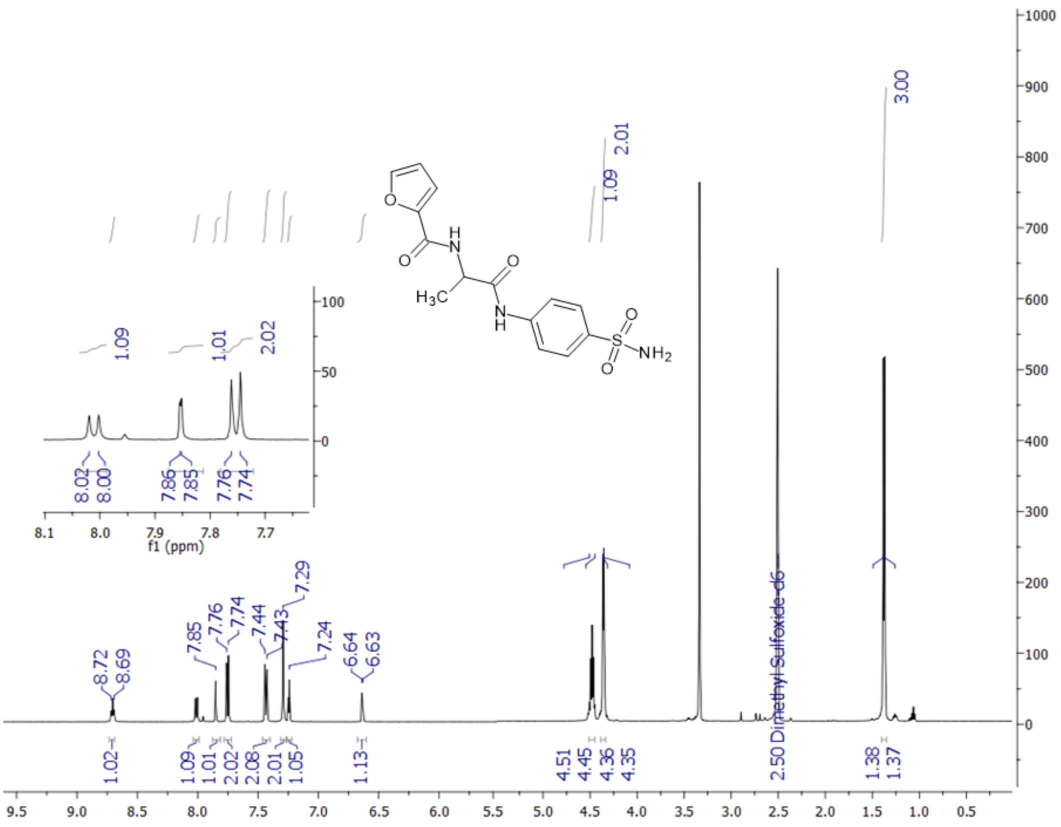

Figure S27. ^{13}C-NMR (126 MHz, DMSO-d_6)) of (*S*)-N-(1-oxo-1-((4-sulfamoylbenzyl)amino)propan-2-yl)furan-2-carboxamide (**40**)

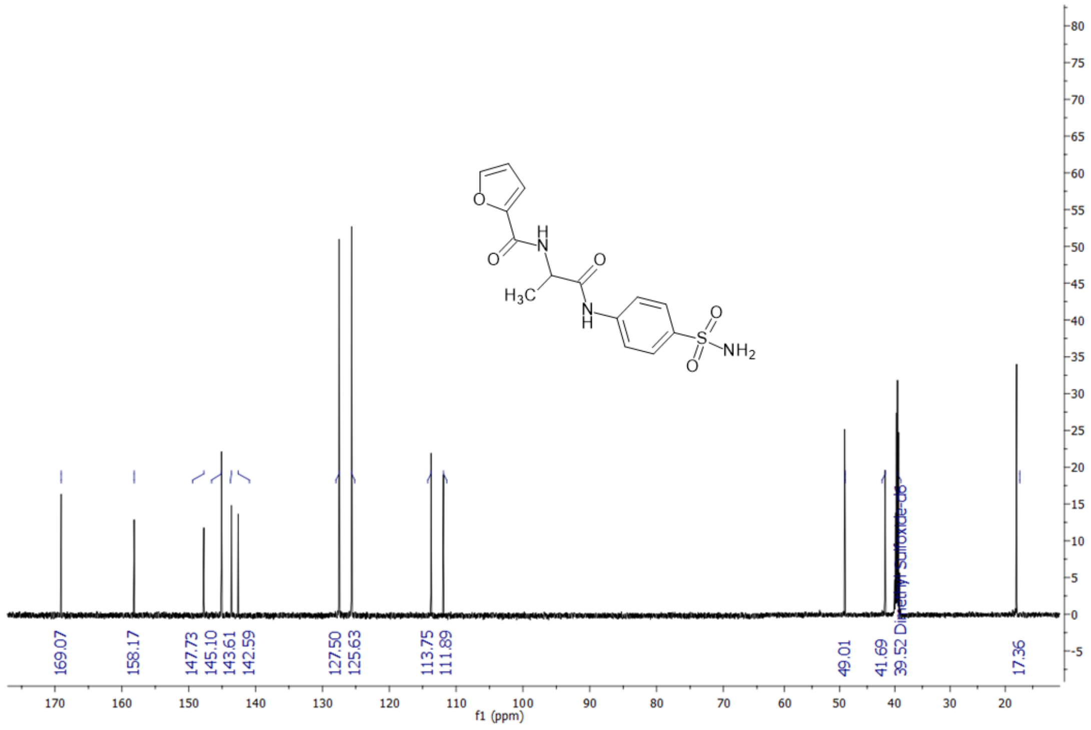

Figure S28. ^{1}H-NMR (500 MHz, DMSO-d_6) of (*S*)-N-(3-methyl-1-oxo-1-((4-sulfamoylbenzyl)amino)butan-2-yl)furan-2-carboxamide, (**41**)

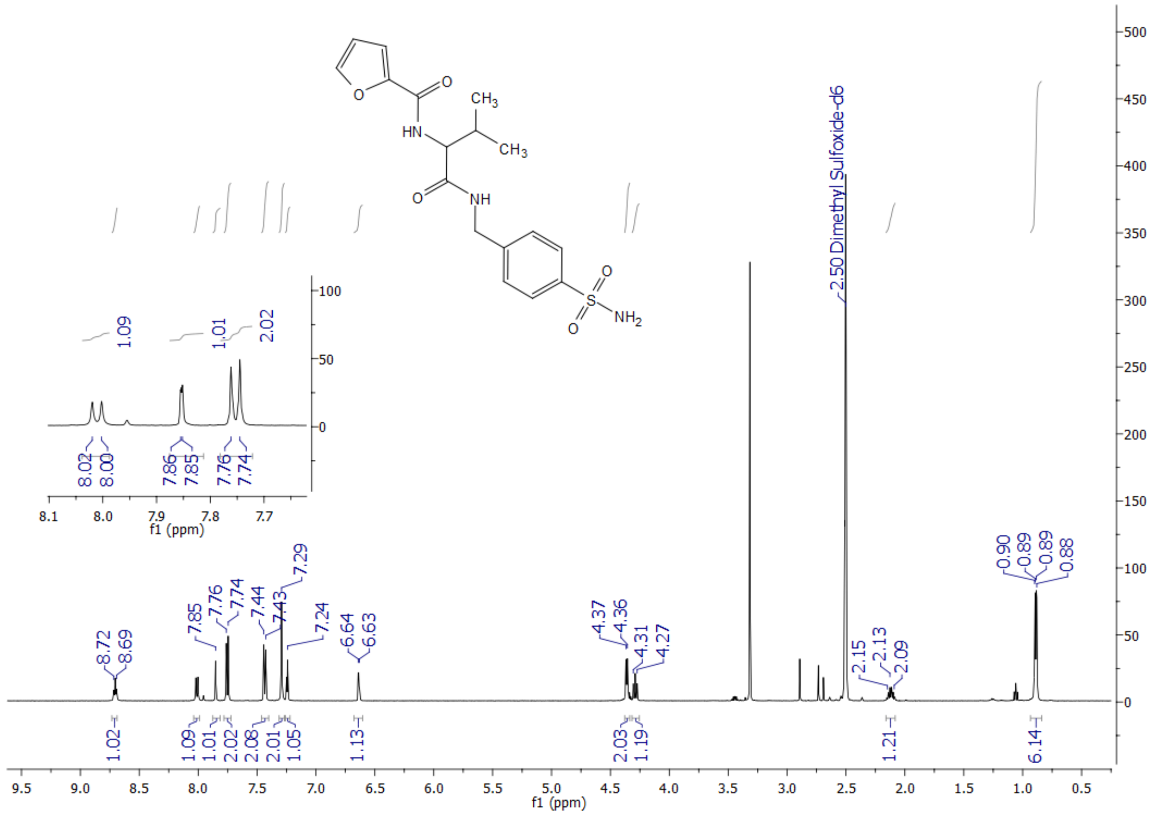

Figure S29. ^{13}C-NMR (126 MHz, DMSO-d_6) of (*S*)-N-(3-methyl-1-oxo-1-((4-sulfamoylbenzyl)amino)butan-2-yl)furan-2-carboxamide, (**41**)

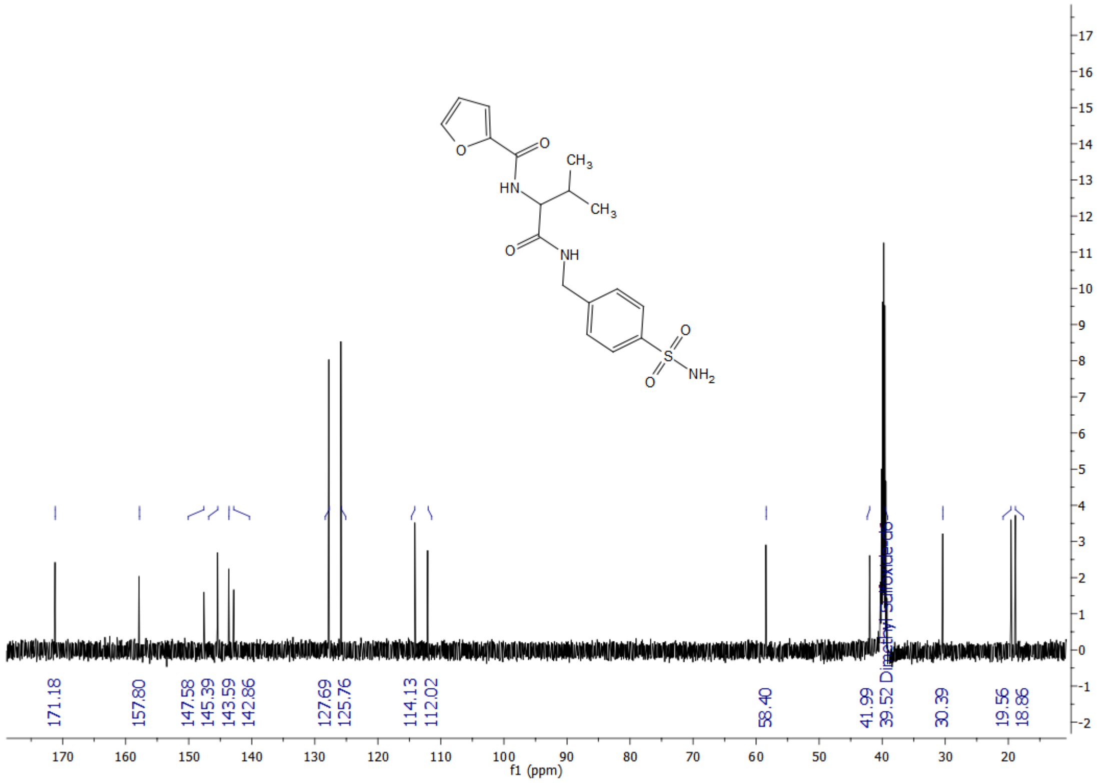

Figure S30. ^{1}H-NMR (500 MHz, DMSO-d_6) of N-(2-oxo-2-((4-sulfamoylbenzyl)amino)ethyl)thiophene-2-carboxamide, (**42**)

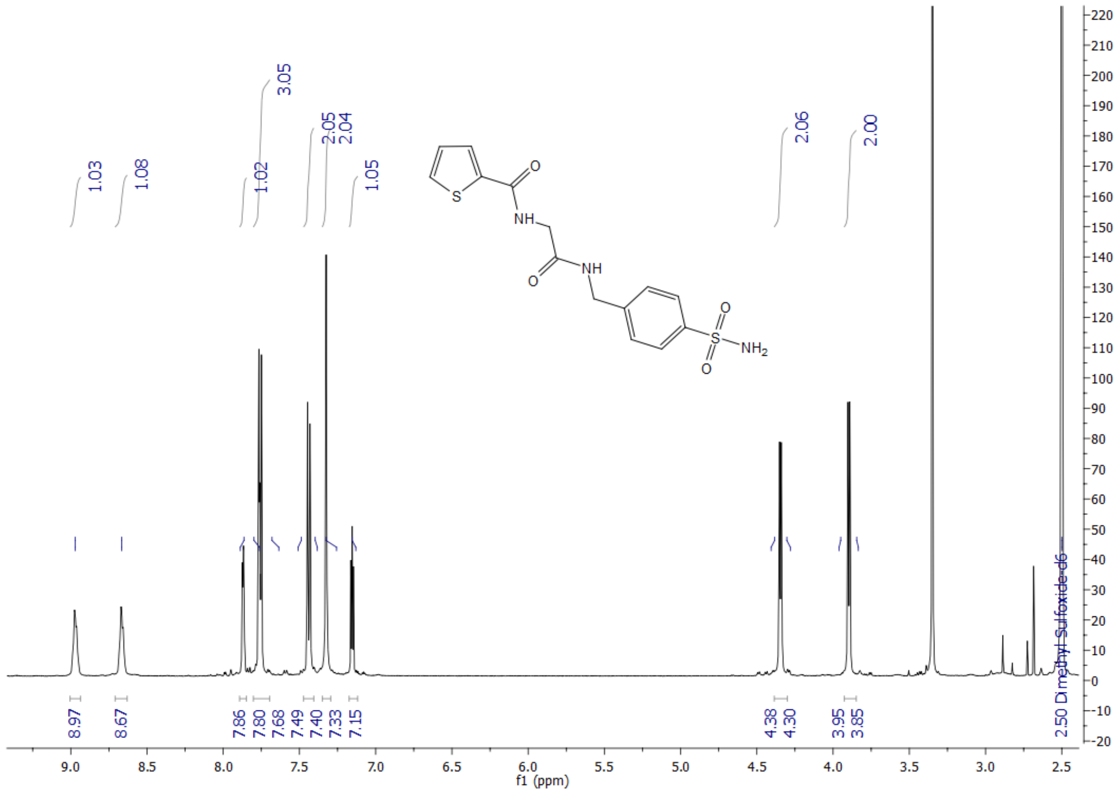

Figure S31. ^{13}C-NMR (126 MHz, DMSO-d_6) of N-(2-oxo-2-((4-sulfamoylbenzyl)amino)ethyl)thiophene-2-carboxamide, (**42**)

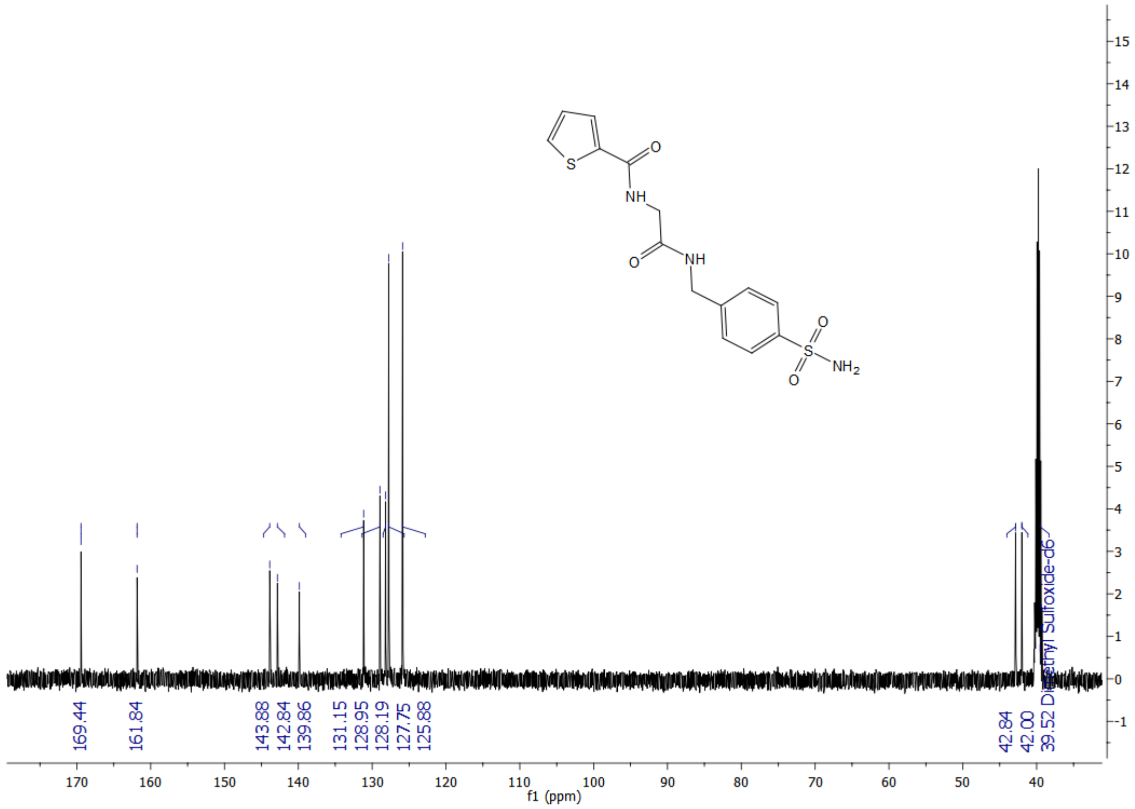

Figure S32. ^{1}H-NMR (500 MHz, DMSO-*d*$_6$) of (*S*)-N-(1-oxo-1-((4-sulfamoylbenzyl)amino)propan-2-yl)thiophene-2-carboxamide, (**43**)

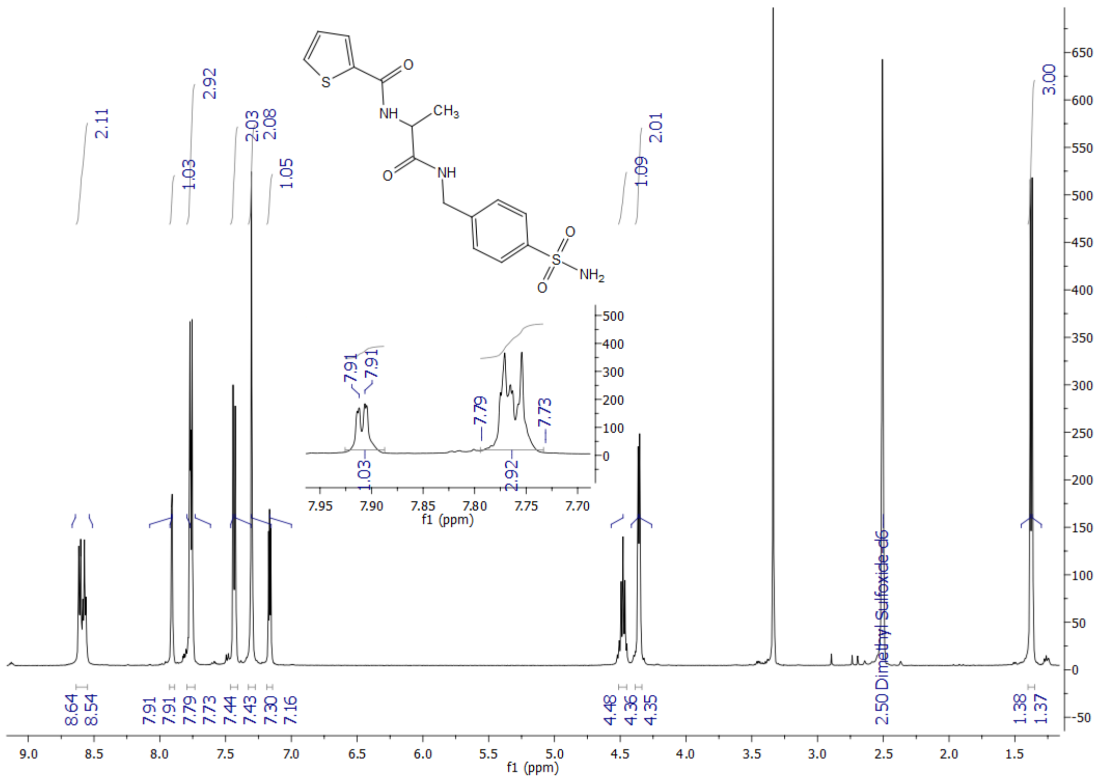

Figure S33. ^{13}C-NMR (126 MHz, DMSO-*d*$_6$) of (*S*)-N-(1-oxo-1-((4-sulfamoylbenzyl)amino)propan-2-yl)thiophene-2-carboxamide, (**43**)

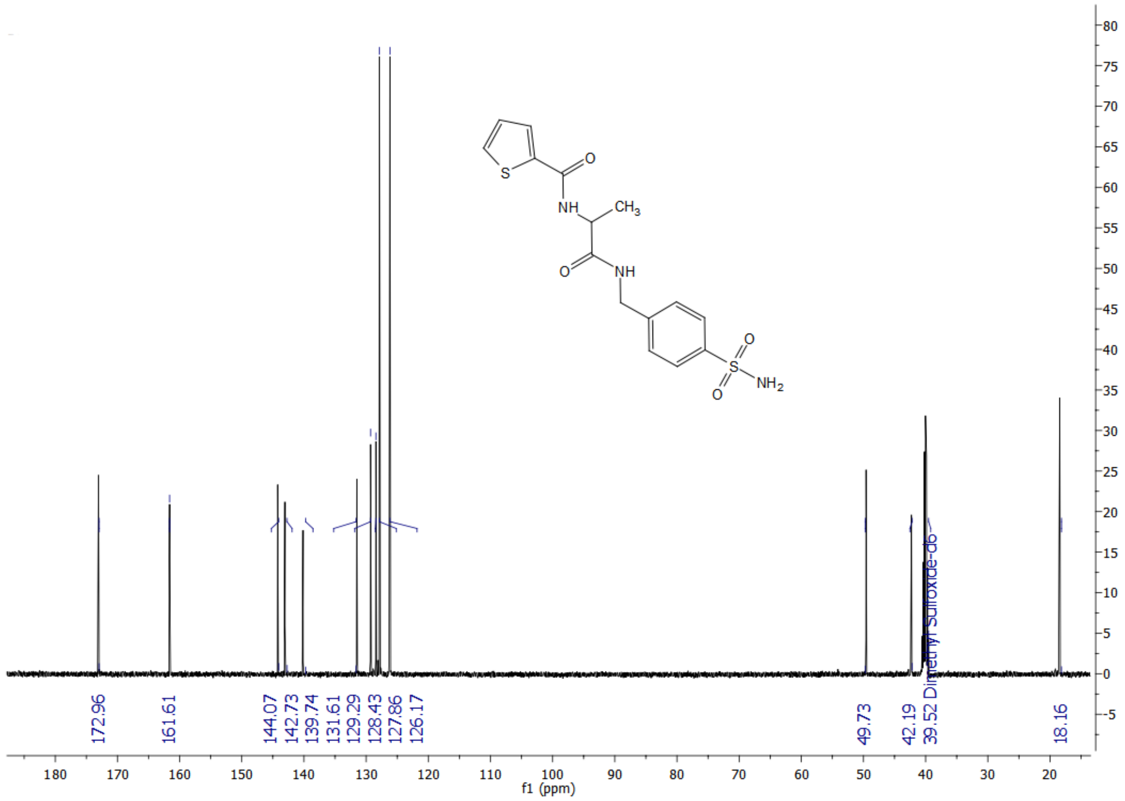

Figure S34. ^{1}H-NMR (500 MHz, DMSO-*d6*) of (*S*)-N-(3-methyl-1-oxo-1-((4-sulfamoylbenzyl)amino)butan-2-yl)thiophene-2-carboxamide, (**44**)

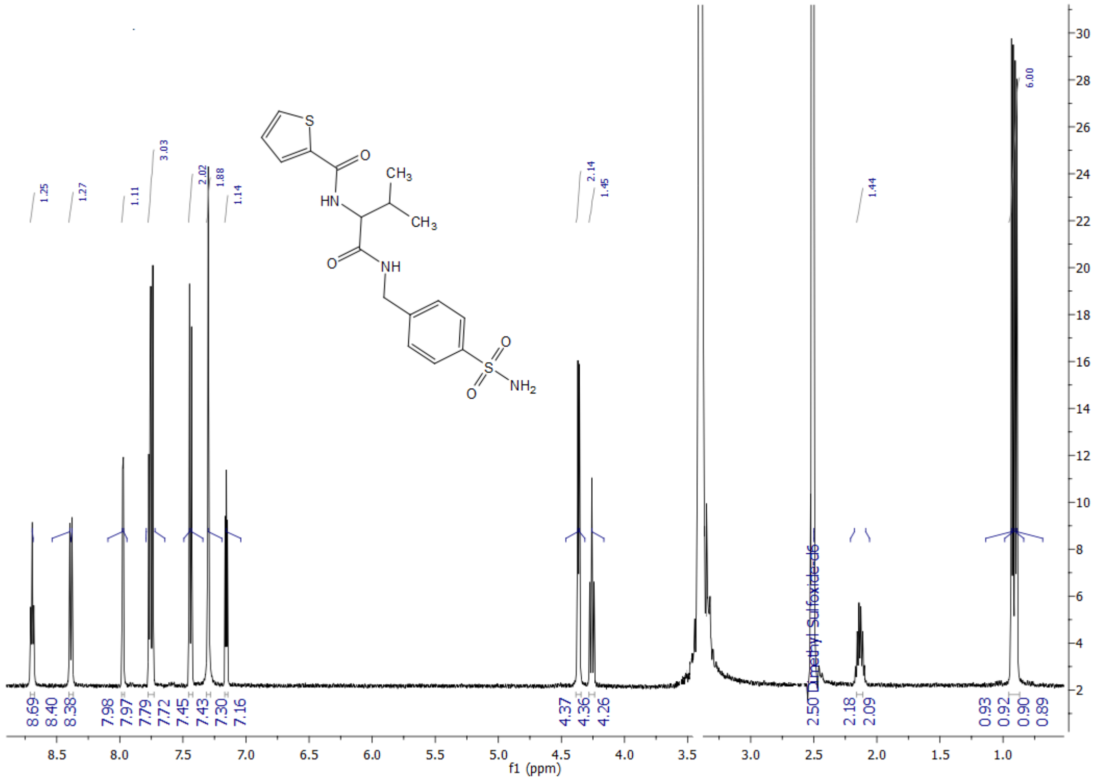

Figure S35. ^{13}C-NMR (126 MHz, DMSO-*d6*) of (*S*)-N-(3-methyl-1-oxo-1-((4-sulfamoylbenzyl)amino)butan-2-yl)thiophene-2-carboxamide, (**44**)

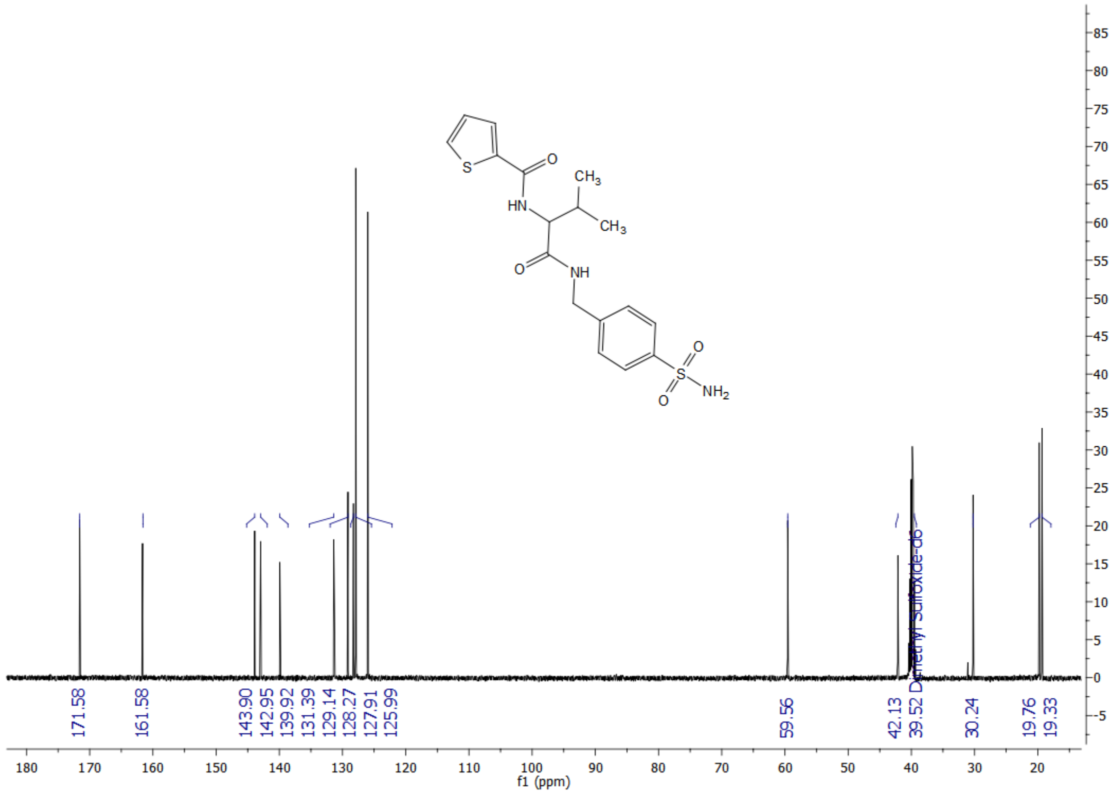

171.58
161.58
143.90
142.95
139.92
131.39
129.14
128.27
127.91
125.99
59.56
42.13
39.52 Dimethyl sulfoxide-d6
30.24
19.76
19.33
f1 (ppm)

2. Selected HPLC Chromatogram and mass spectra

HPLC-MS measurements for compounds **7-9**, **11-12**, **14-15** were performed on an LCMS-2020 system from Shimadzu equipped with a Luna® C18(2) column (3µm, 100A, 100 × 4.6 mm) using a linear gradient of CH_3CN + 0.05% HCOOH (5→95% in 9 min) in H_2O + 0.05% HCOOH at a flow rate of 1.0 mL/min.

Figure S36. HPLC chromatogram for compound (9H-fluoren-9-yl)methyl (2-oxo-2-((4-sulfamoylphenyl)amino)ethyl)carbamate, (**7**)

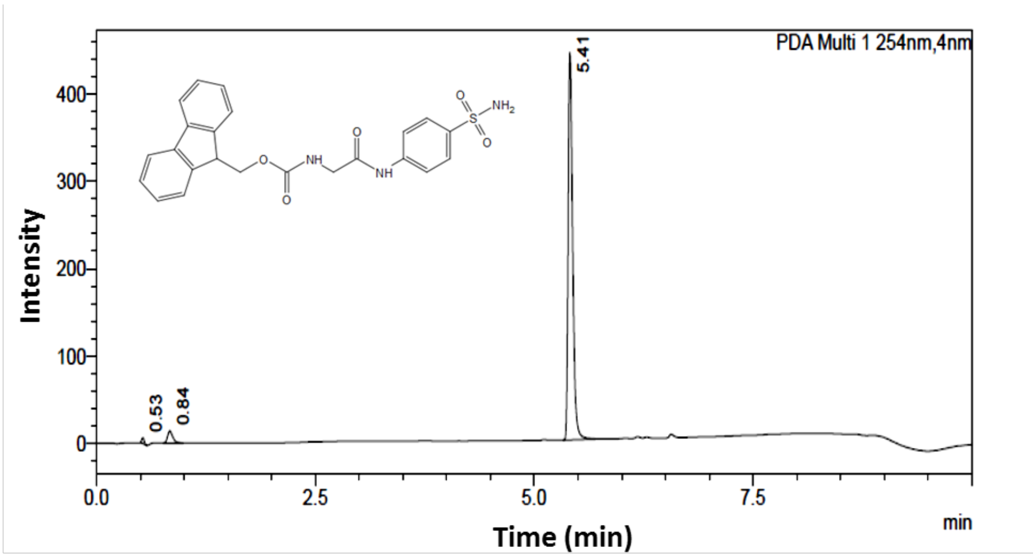

Figure S37. HPLC chromatogram for compound (9*H*-fluoren-9-yl)methyl (1-oxo-1-((4-sulfamoylphenyl)amino)propan-2-yl)carbamate, (**8**)

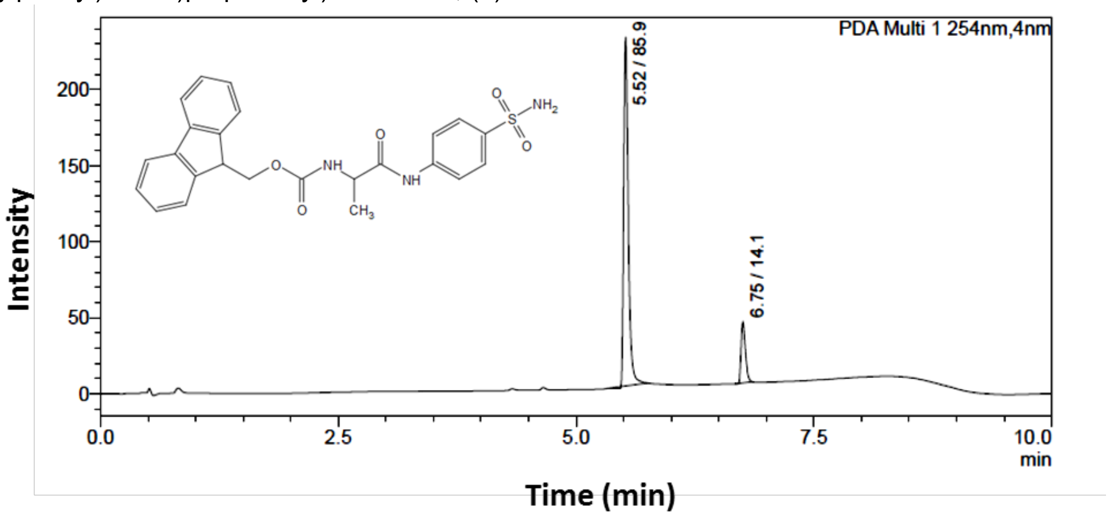

Figure S38. HPLC chromatogram for compound (9*H*-fluoren-9-yl)methyl (3-methyl-1-oxo-1-((4-sulfamoylphenyl)amino)butan-2-yl)carbamate, (**9**)

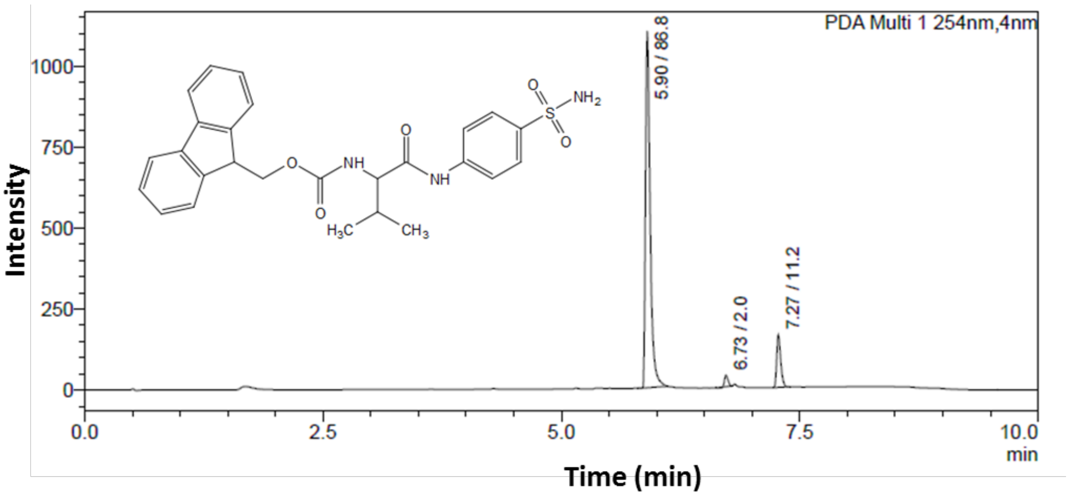

Figure S39. HPLC chromatogram for compound 2-amino-N-(4-sulfamoylphenyl)propanamide, (**11**)

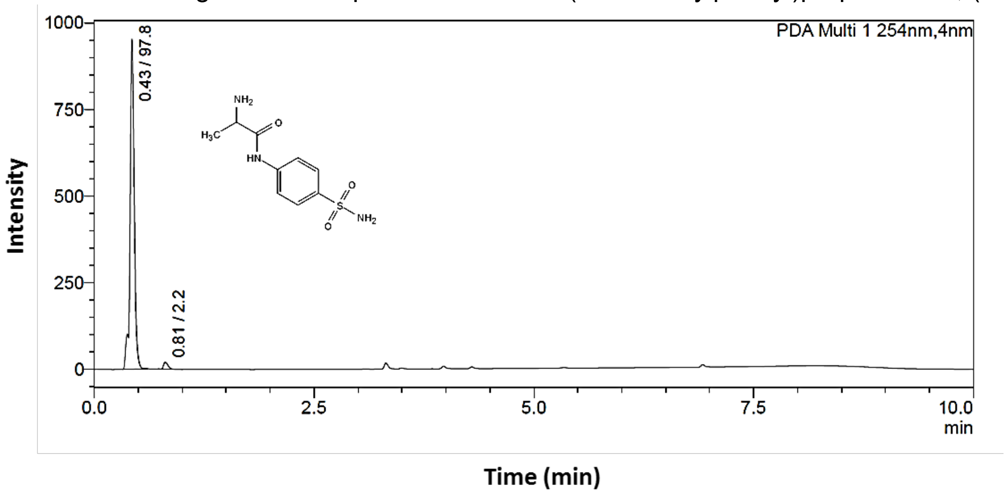

Figure S40. HPLC chromatogram for compound 2-amino-3-methyl-N-(4-sulfamoylphenyl)butanamide, (**12**)

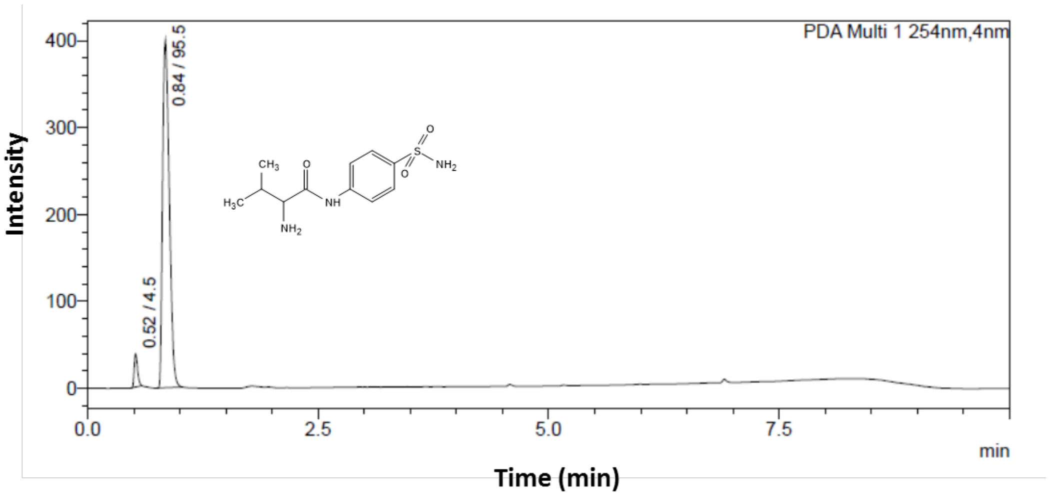

Figure S41. HPLC chromatogram for compound N-(1-oxo-1-((4-sulfamoylphenyl)amino)propan-2-yl)benzamide, (**14**)

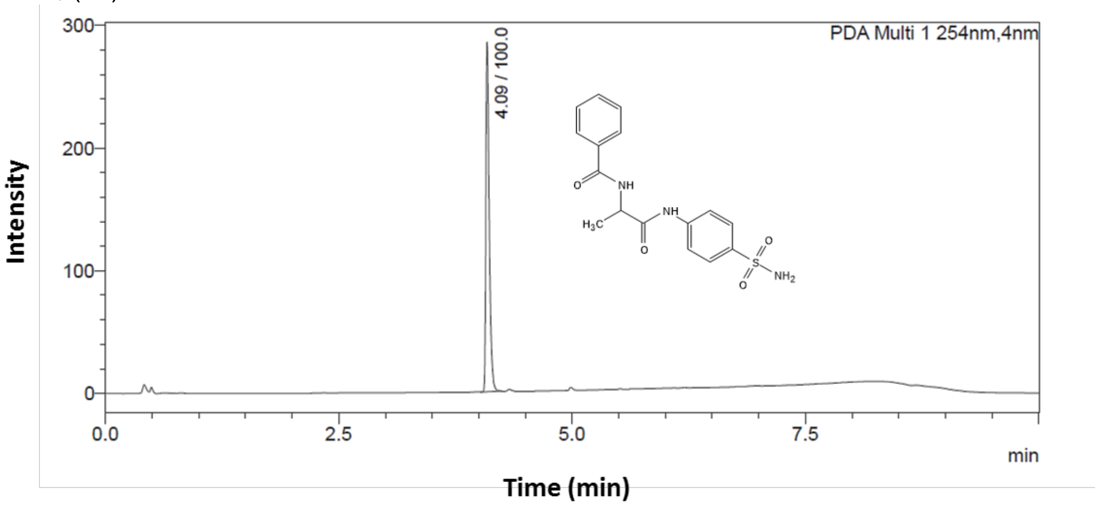

Figure S42. HPLC chromatogram for compound N-(3-methyl-1-oxo-1-((4-sulfamoylphenyl)amino)butan-2-yl)benzamide, (**15**)

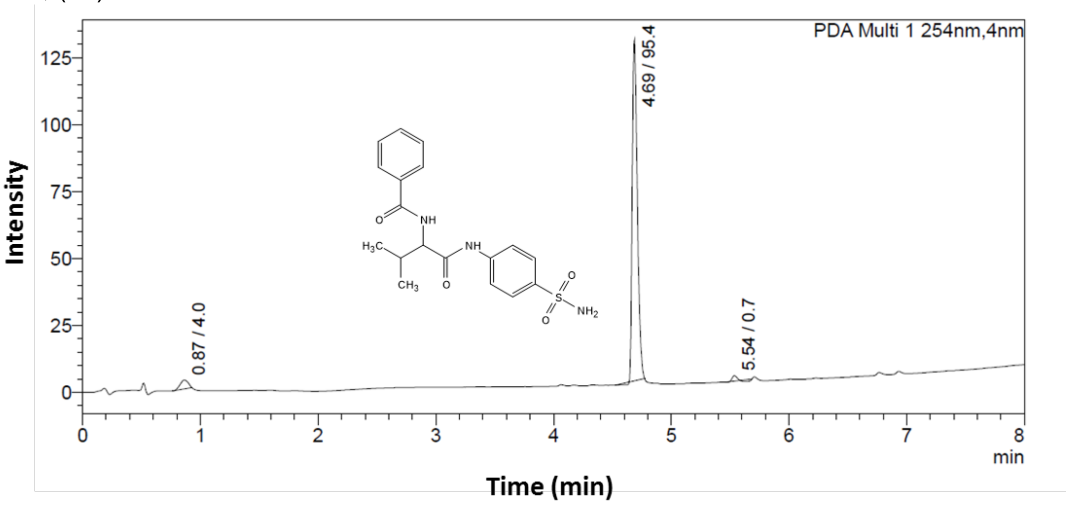

3. Sequence alignment of targeted CA enzymes

```
P00915      CAH1_HUMAN         1    -----------------MASPDWGYDD-KNGPEQWSKLYPIA-NGNN--QSPVDIKTSET    39
P00918      CAH2_HUMAN         1    ------------------MSHHWGYGK-HNGPEHWHKDFPIA-KGER--QSPVDIDTHTA    38
A0A0H6VI20  A0A0H6VI20_VIBCL   1    MKKTTWVLAMVASMSFGVQASEWGYEG-EHAPEHWGKVAPLCAEGKN--QSPIDVAQSV-    56
A0A086SLX8  A0A086SLX8_VIBCL   1    ---------MPEIKQLFENNSKWSESIKAETPEYF----AKLAKGQNPDFLWIGCADSRV    47
A0A085T4G3  A0A085T4G3_VIBCL   1    -----------------MMSSIRSYKG-I---------VPKLGEGVYIDSSAVLVGDIEL    33
                                                           .                   :*       :

P00915      CAH1_HUMAN        40    KHDTSLKPISVSYNPATAKE-----IINVGHSFHVNFEDNDNR---SVLKGG--PFSDSY    89
P00918      CAH2_HUMAN        39    KYDPSLKPLSVSYDQATSLR-----ILNNGHAFNVEFDDSQDK---AVLKGG--PLDGTY    88
A0A0H6VI20  A0A0H6VI20_VIBCL  57    --EADLQPFTLNYQG-QVVG-----LLNNGHTLQAIVRGNNPL----QI------DGKTF    98
A0A086SLX8  A0A086SLX8_VIBCL  48    P----AERLTGLYSGELFVH--------RNVANQVIHTDLNCL---SVVQYAVDVLQVKH    92
A0A085T4G3  A0A085T4G3_VIBCL  34    GDDASIWPLVAARGDVNHIRIGKRTNIQDGSVLHVTHKNAENPNGYPLCIGDDVTIGHKV    93
                                            :                    .   :.   . :                 .

P00915      CAH1_HUMAN        90    RLFQFHFHWGSTNEHGSEHTVDGVKYSA----ELHVAHW--NSAKYSSLAEAASKADGLA    143
P00918      CAH2_HUMAN        89    RLIQFHFHWGSLDGQGSEHTVDKKKYAA----ELHLVHW--NT-KYGDFGKAVQQPDGLA    141
A0A0H6VI20  A0A0H6VI20_VIBCL  99    QLKQFHFHTPS------ENLLKGKQFPL----EAHFVHA--D-----------EQGNLA    134
A0A086SLX8  A0A086SLX8_VIBCL  93    IIVCGHYGCG-----GVTAAIDNPQLGLINNWLLHIRDYYLKHREYLDQMPAEDRSDKLA    147
A0A085T4G3  A0A085T4G3_VIBCL  94    MLHGCTIHDRVLVGMGSI-VLDG----------------------------------AVI    118
                                     :                  :.                                    :

P00915      CAH1_HUMAN       144    VIGVLMKVGEANPKLQKVLDALQAIKTKGKRAPFTN-FDPSTLLPSSLDFWTYPGSLTHP    202
P00918      CAH2_HUMAN       142    VLGIFLKVGSAKPGLQKVVDVLDSIKTKGKSADFTN-FDPRGLLPESLDYWTYPGSLTTP    200
A0A0H6VI20  A0A0H6VI20_VIBCL 135    VVAVMYQVGSENPLLKVLTA---DMPTKGNSTQLTQGIPLADWIPESKHYYRFNGSLTTP    191
A0A086SLX8  A0A086SLX8_VIBCL 148    EINVAEQVYNLANS-----TVLQNAWERGQAVEV-----------HGFVYGIEDGRLEY-    190
A0A085T4G3  A0A085T4G3_VIBCL 119    ENDVMIGAGSLVPPGKR--------------------------LESGFLYMGSPVKQA-R    151
                                       :   . .                                    .  :

P00915      CAH1_HUMAN       203    PLYESVTWIICKESISVSSEQLAQFRSLLSNVEGDNAVPMQHNNRPTQPLKGRTVRASF-    261
P00918      CAH2_HUMAN       201    PLLECVTWIVLKEPISVSSEQVLKFRKLNFNGEGEPEELMVDNWRPAQPLKNRQIKASFK    260
A0A0H6VI20  A0A0H6VI20_VIBCL 192    PCSEGVRWIVLKEPAHLSNQQEQQL-----------SAVMGHNNRPVQPHNARLVLQAD-    239
A0A086SLX8  A0A086SLX8_VIBCL 191    ---LGVR---CASRSAVEDNYHKALE-----------KIL----NPNHRLLCR-------    222
A0A085T4G3  A0A085T4G3_VIBCL 152    PLNDKERAFLVKSSSNYVQSKNDYL----NDV--------------------KTVRE---    184
                                                .     ..    :                           :
```

Alignment of hCA I (P00915), hCA II (P00918), VchCA α (A0A0H6VI20), VchCA β (A0A086SLX8) and VchCA γ (A0A085T4G3) performed by using the online platform Uniprot (https://www.uniprot.org/). The residues of the metal binding site for the three available CA crystal structures are highlighted in pink.
